# Rules and mechanisms governing G protein coupling selectivity of GPCRs

**DOI:** 10.1016/j.celrep.2023.113173

**Published:** 2023-09-23

**Authors:** Ikuo Masuho, Ryoji Kise, Pablo Gainza, Ee Von Moo, Xiaona Li, Ryosuke Tany, Hideko Wakasugi-Masuho, Bruno E. Correia, Kirill A. Martemyanov

**Affiliations:** 1 Department of Neuroscience, UF Scripps Biomedical Research, Jupiter, FL 33458, USA; 2 Pediatrics and Rare Diseases Group, Sanford Research, Sioux Falls, SD 57104, USA; 3 Department of Pediatrics, Sanford School of Medicine, University of South Dakota, Sioux Falls, SD 57105, USA; 4 Laboratory of Protein Design and Immunoengineering, School of Life Sciences, École Polytechnique Fédérale de Lausanne and Swiss Institute of Bioinformatics, Lausanne, Switzerland; 5 Lead contact

## Abstract

G protein-coupled receptors (GPCRs) convert extracellular stimuli into intracellular signaling by coupling to heterotrimeric G proteins of four classes: G_i/o_, G_q_, G_s_, and G_12/13_. However, our understanding of the G protein selectivity of GPCRs is incomplete. Here, we quantitatively measure the enzymatic activity of GPCRs in living cells and reveal the G protein selectivity of 124 GPCRs with the exact rank order of their G protein preference. Using this information, we establish a classification of GPCRs by functional selectivity, discover the existence of a G_12/13_-coupled receptor, G_15_-coupled receptors, and a variety of subclasses for G_i/o_-, G_q_-, and G_s_-coupled receptors, culminating in development of the predictive algorithm of G protein selectivity. We further identify the structural determinants of G protein selectivity, allowing us to synthesize non-existent GPCRs with *de novo* G protein selectivity and efficiently identify putative pathogenic variants.

## In brief

Masuho et al. measured the kinetics of GPCR enzymatic activity, revealing ranks of G protein selectivity for 124 GPCRs. This led to development of GPCRs’ functional classification, identification of distinct G protein couplings, algorithms for predicting G protein selectivity, *de novo* design of GPCRs, and identification of functionally biased GPCR variants.

## Graphical abstract

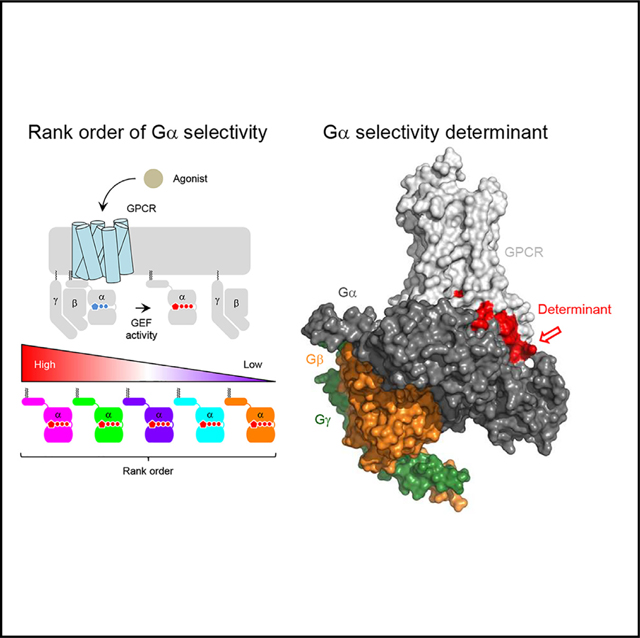

## INTRODUCTION

G protein-coupled receptors (GPCRs) comprise the largest gene family in the human genome with more than 800 members.^1–3^ GPCRs are critically involved in a wide range of physiological functions including development, immunity, hormonal regulation, and neuronal activity.^4–10^

Canonically, GPCRs transduce the extracellular stimuli into intracellular reactions by activating heterotrimeric G proteins consisting of Gα, Gβ, and Gγ subunits.^11^ Ligand-bound GPCRs act as guanine nucleotide exchange factors (GEFs), catalyzing the exchange of GDP to GTP on Gα subunits,^11^ triggering the dissociation of GTP-bound Gα from Gβγ dimer. Both Gα-GTP and free Gβγ are active forms of G proteins that can bind to and regulate the activity of various effector molecules.

GPCRs respond to a broad range of extracellular stimuli evoking complex intracellular reactions.^12^ This diversity of signaling reactions initiated by GPCRs is underscored by the diversity of G proteins they activate. The human genome encodes 16 Gα subunits grouped into four subfamilies, Gα_i/o_, Gα_q_, Gα_s_, and Gα_12/13_.^13^ Importantly, individual G proteins initiate qualitatively different signaling cascades. Thus, the identity of G proteins activated by GPCRs determines downstream signaling events and, in turn, cellular responses. The issue of G protein selectivity of GPCRs is therefore central to understanding the biology of these signaling systems.

Historically, the majority of GPCRs were thought to couple only to a single type of G protein.^14^ However, this picture is rapidly changing, and most GPCRs have been documented to engage multiple G proteins.^15–20^ This multi-valent G protein coupling is likely responsible for the vast capacity of GPCRs to program complex cellular responses. Signaling via different G proteins may also occur with different timing, further endowing GPCRs with the ability to activate various cascades in waves.^15,21^ However, temporal aspects of G protein selectivity, relative efficiencies of different GPCR-G protein pairings, and their physiological relevance are not well understood.

It is pervasive in the field to define GPCRs by the single preferred G protein they activate, so-called primary coupling, functionally classifying GPCRs as Gα_i/o_-, Gα_q_-, Gα_s_-, or Gα_12/13_-coupled receptors. However, given that GPCRs couple to multiple G proteins, establishing primary coupling with certainty may not be straightforward. Accordingly, recent attempts to systematically compare G protein coupling specificity of GPCRs^15–18,20,22^ resulted in low agreement across individual studies.^23^

In this study, we employed a fundamentally different approach from prior efforts to evaluate the G protein selectivity of a large number of GPCRs. We quantitatively determined the GEF activity of GPCRs toward G proteins using kinetic measurements with a bioluminescence resonance energy transfer (BRET) strategy that allows parallel comparison of unmodified Gα in real time.^15,24^ This resulted in a fine-grained view of G protein selectivity for 124 mammalian GPCRs, allowing us to classify them according to rank order of G protein preferences. With this information, we developed an algorithm for predicting the G protein selectivity of GPCRs, interrogated the structural basis of the selectivity, designed synthetic GPCRs with novel specificity, and analyzed the impact of genetic variants on GPCR-G protein selectivity.

## RESULTS

### A quantitative kinetic approach to establish rank order selectivity of G protein activation by GPCRs

**Figure 1.**
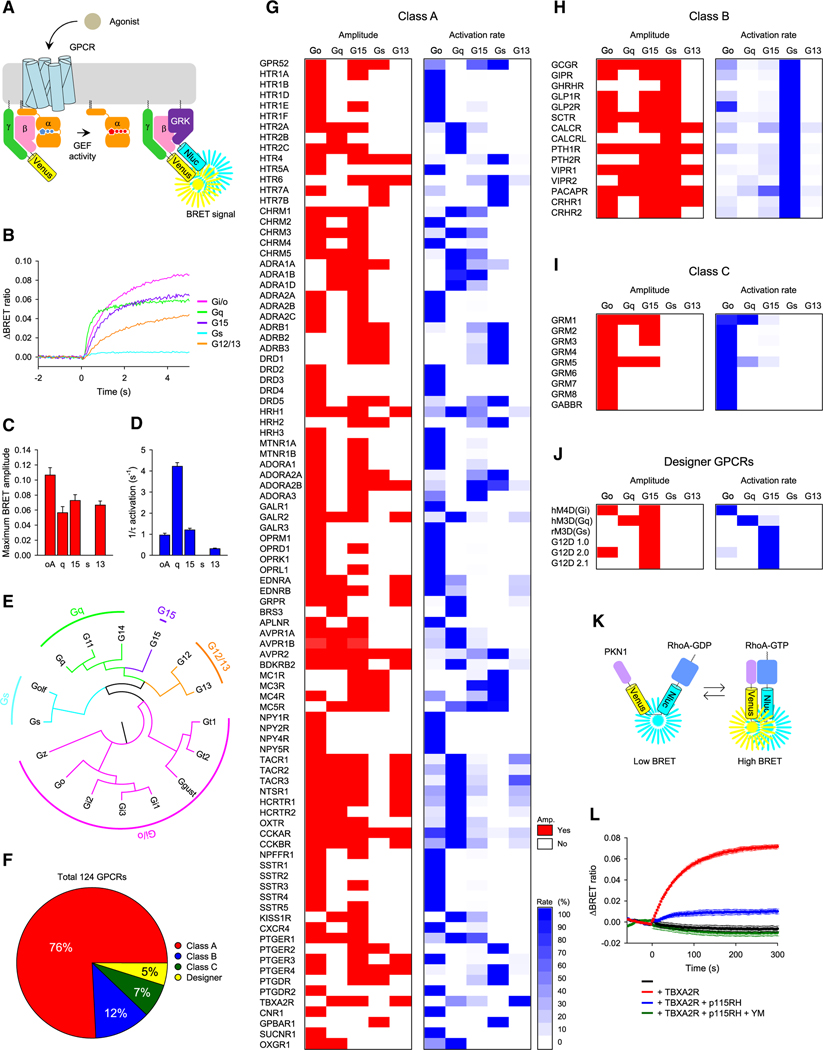
Characterization of Gα-selectivity of 124 GPCRs with *in cellulo* biochemical assay (A) Schematic representation of the BRET-based kinetic assay for real-time monitoring of G protein activity. (B–D) Examples of data obtained by the BRET assay. (E) Phylogenetic tree of human Gα subunits. (F) The number and percentage of GPCRs used in this study. (G–J) Comprehensive analysis of G protein selectivity of GPCRs induced by saturating concentrations of endogenous agonists. By taking the fastest reaction as 100%, the G protein activation rates that can be activated by each GPCR were compared. All agonists used in this study are listed in Table S1 with their names and concentrations. (K) Schematic representation of the BRET-based kinetic assay for real-time monitoring of RhoA activity. (L) Coupling of TBXA2R to endogenous G_12/13_. A minimum of three independent experiments were performed. The mean and SEM values are shown in the bar graphs and reported in Table S1.

To quantitatively examine the G protein selectivity of GPCRs, we employed the BRET strategy that measures G protein activation by fast kinetic monitoring in living cells (Figure 1A). In this assay, stoichiometric trimer formation, subcellular localization, and equivalent expression levels of exogenous G proteins were extensively optimized to ensure the specificity of signal detection.^15^

The first key advantage of this assay is the ability to compare the behavior of native Gα subunits by a common readout: the release of Gβγ subunits upon G protein activation. The second critical feature lies in the direct measurement of G protein activation rates upon GPCR stimulation. From an enzymological perspective, enhanced catalytic efficiency, due to better substrate recognition, increases the reaction rate, making onset kinetics (*k*_ON_) of G protein activation a true measure of selectivity.

Importantly, in this assay, increasing concentrations of agonists or GPCR expression levels have been shown to correlate with activation rates,^25^ indicating that the G protein activation rate observed in this assay is primarily determined by the GEF activity of GPCRs. Moreover, we confirmed that even if the agonist concentration (Figures S1A–S1C) or the expression level (Figures S1D and S1E) of GPCR is different, the rank order of its G protein selectivity evaluated kinetically remains invariable, indicating that, as long as G proteins can be activated, agonist concentrations and receptor expression levels do not alter the rank order of G protein selectivity. Nevertheless, to ensure the detection of all potential G protein activations, all GPCRs were stimulated with saturated concentrations of endogenous agonists (see Table S1 for details).

### G protein selectivity profiles for 124 GPCRs

To test our strategy, we examined the G protein-coupling profile of the promiscuous cholecystokinin 2 receptor (CCK_2_R) (*CCKBR*) (Figure 1B). Analysis of the response amplitudes shows that CCK_2_R can activate several G proteins belonging to four G protein groups: Gα_i/o_, Gα_q_, Gα_15_, and Gα_12/13_ with relatively minor differences but not G_s_ (Figure 1C). In contrast, analysis of the activation rates clearly delineated the rank order of G protein activation with Gα_q_ coupling being the best, followed by Gα_i/o_, Gα_15_, and Gα_12/13_ (Figure 1D). Thus, kinetic measurements allow detecting substantial differences in activation of individual G proteins (Figures 1B and 1D), while the amplitude-based estimates often obscure these differences (Figures 1B and 1C).

While the assay is capable of monitoring individual behavior of all Gα subunits,^15,26^ in this study, we focused on examining the inter-class differences by selecting one representative Gα from each of the four classes of G proteins (Gα_oA_, Gα_q_, Gα_sS_, and Gα_13_) and an outlier Gα_15_ (Figure 1E). Since Gβ and Gγ subunits do not significantly affect G protein selectivity,^27^ Gβ_1_ and Gγ_2_ were chosen as a ubiquitously used model.

With these five Gα subunits, we examined G protein-coupling profiles of 124 GPCRs across three major classes, A (94), B (15), and C (9) as well as six synthetic GPCRs (Figures 1G–1J). Experimental conditions were optimized; e.g., serotonin receptors were assessed under serum starvation conditions, which significantly enhanced the response (Figures S1F–S1H). All experimental conditions for the evaluation of individual receptors are listed in Table S1. Our strategy was to first measure maximal response amplitude to establish whether coupling occurs in principle. In case statistically significant activation was detected in comparison with control experiments without GPCR or Gα, we determined *k*_ON_ to quantify the G protein selectivity of GPCRs and established a rank order (Figures 1G–1J; Table S1).

### Insights from the analysis of primary coupling of GPCRs

G protein coupling of many GPCRs has been extensively studied in the past using a variety of methods. This knowledge has been cataloged by two databases: the IUPHAR/BPS Guide to Pharmacology (GtoPdb) (https://www.guidetopharmacology.org/)^28^ and the G protein database (Gproteindb; https://GproteinDb.org).^23^ The GtoPdb inventories manually curated information on the G protein selectivity of GPCRs, and the Gproteindb logs information on GPCR-G protein coupling obtained from GtoPdb and two large-scale screening efforts.^23^ Thus, before in-depth analysis of the patterns we observed, we first compared our dataset with the information in these databases.

**Figure 2.**
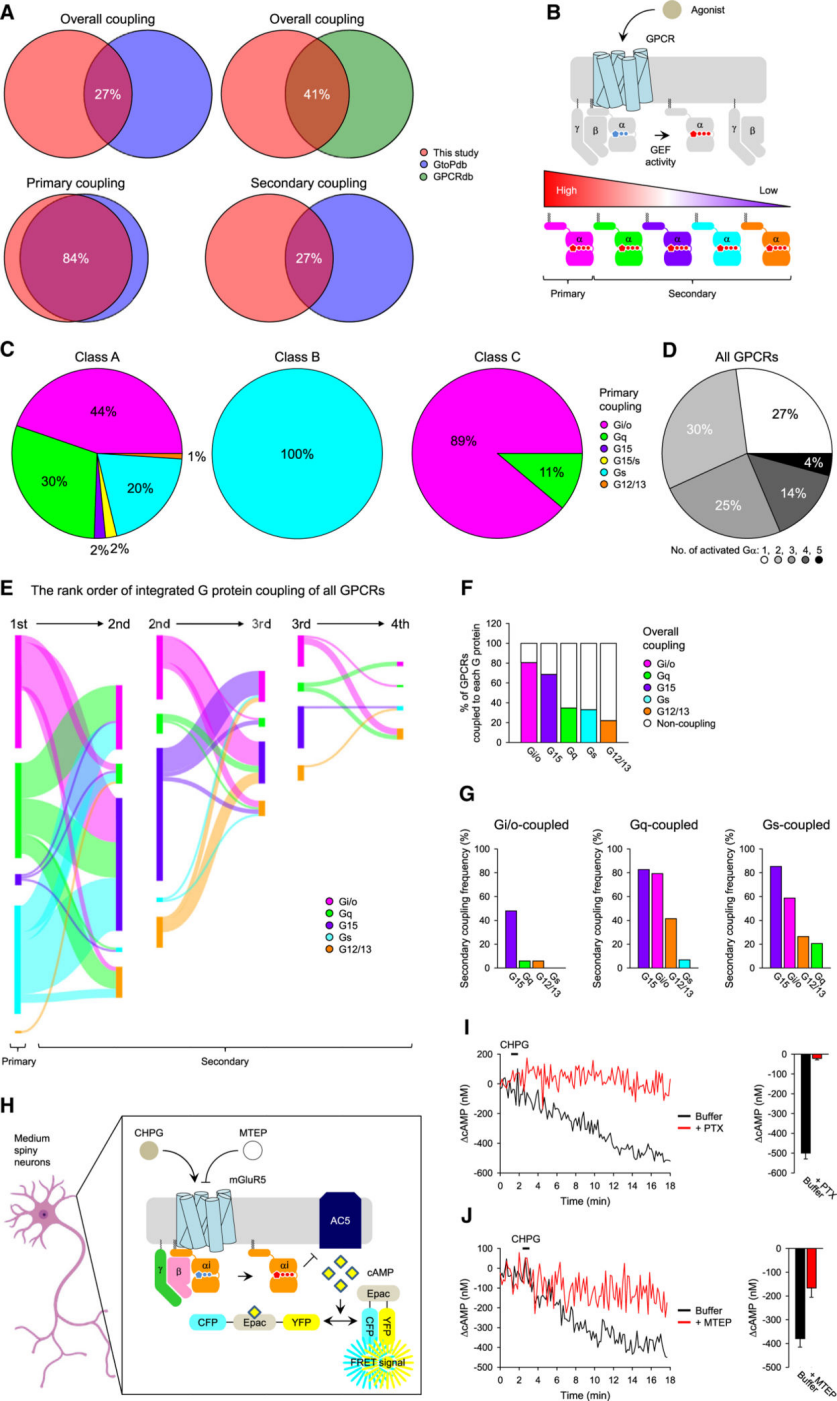
Characterization and classification of GPCRs by Gα selectivity and promiscuity (A) Comparison of G protein selectivity reported in GtoPdb, GProteindb, and our study. (B) The rank order of Gα selectivity by the strength of GEF activity determined from the G protein activation rates. (C) The primary G protein coupling selectivity of GPCRs by class. (D) Promiscuity of all class GPCRs examined. (E) The rank order of integrated G protein coupling of all GPCRs tested. The width of the lines and nodes indicates the number of couplings. (F) Percentage of GPCRs coupled with each of the five G proteins. (G) Secondary coupling of GPCRs by G_i/o_-, G_q_-, and G_s_-coupled receptors. Designer GPCRs were excluded from the analysis in this figure (A and C–G). (H) Schematic representation of the FRET-based cAMP assay using primary medium spiny neurons cultured from cAMP Encoder Reporter (CAMPER) mice.^59^ (I and J) mGluR5-selective agonist (1 mM CHPG)-induced cAMP dynamics in the presence or absence of PTX (I) (n = 6 neurons) or 10 μM MTEP (J) (n = 6 neurons). The traces are the average values. The mean and SEM are shown in the bar graphs.

When overall GPCR coupling across all G protein classes was considered, we found relatively modest agreement of our dataset with GProteindb and GtoPdb, which did not exceed 50% (Figure 2A). To understand the reasons for this disagreement, we segregated the most preferred Gα substrate, designating it as “primary” from the other G proteins activated by a given GPCR, collectively binning the other G proteins as secondary substrates (Figure 2B).

Considering only the primary coupling of GPCRs dramatically improved agreement between datasets: 84% match compared to GtoPdb (Figure 2A). This observation highlights that the data we obtained accurately capture the G protein coupling of GPCRs and further reinforces the consensus about the main signaling modality for many GPCRs. Interestingly, most disagreements in the primary coupling were with GPCRs that promiscuously couple to several G proteins (Table S1).

Our analysis of primary coupling preferences revealed several interesting patterns of GPCR selectivity (Figures 2C, 2D, S2A, and S2B). For instance, we found that the majority of class A receptors preferred Gα_i/o_, although they also exhibited the greatest G protein coupling diversity (Figure 2C). Class B receptors were the most homogeneous, being represented exclusively by Gα_s_-coupled receptors. Class C receptors were predominantly G_i/o_ coupled but also contained a minor fraction of Gα_q_-coupled receptors.

### Unexpected primary couplings of GPCRs

Mining our dataset revealed several notable observations not documented before. First, we found the receptor that primarily couples to Gα_12/13_. While many receptors (22%) can couple to Gα_13_, only the thromboxane receptor (*TBXA2R*) showed a preference for it as a primary substrate, making it the only Gα_12/13_-coupled receptor currently known. This observation was confirmed with a BRET-based RhoA sensor as a readout to G_12/13_ coupling of TBXA2R (Figure 1L).

Second, we found a group of receptors primarily coupled to Gα_15_, which was not previously known to exist. These are represented by adenosine A_2B_ (*ADORA2B*) and A_3_ (*ADORA3*) receptors, previously classified as Gα_s_- and Gα_i/o_-coupled receptors, respectively. Because of unexpected G protein selectivity, we confirmed their G_15_ coupling by expressing PTX and by treating cells with YM-254890^29^ (Figures S1I–S1M). Melanocortin 3 (*MC3R*) and 5 (*MC5R*) receptors also couple primarily to Gα_15_, although they also show comparable activity on Gα_s_. These G_15_ couplings were further confirmed by a-MSH-induced intracellular Ca^2+^ elevation in the presence of YM-254890 and pertussis toxin (PTX) (Figures S1N and S1O).

Interestingly, with the exception of MC5R, these receptors do not couple to Gα_q_ despite the homology of Gα_15_ to Gα_q_ proteins (Figure 1G; Table S1). Furthermore, the majority of GPCRs that can couple to Gα_15_ did not activate Gα_q_ (Figure 1G; Table S1). These observations indicate that Gα_15_ has distinct GPCR recognition principles and, given its unique biology,^30,31^ needs to be treated separately.

Third, our analysis significantly revises the G protein selectivity of synthetic receptors designed for chemogenetic manipulations (Figure 1J; Table S1). We have confirmed that hM4R(Gi) and hM3D(Gq) indeed primarily couple to Gα_i/o_ and Gα_q_, as designed. However, the rM3D(Gs), which was designed to activate G_s_, had weak but reliable activity on this G protein, which did not reach the statistical significance threshold we set for positively identifying coupling activity even after optimizations (Figure S1P). Instead, rM3D(Gs) produced strong activation of Gα_15_, which essentially classifies it as a Gα_15_-coupled receptor (Figure S1Q). We were further unable to confirm the primary G protein selectivity of G12D receptors designed to activate Gα_12_ exclusively.^16^ We did not detect significant activation of either Gα_12_ or Gα_13_ by any of the three versions of G12D (Figure S1R). Instead, this receptor powerfully activated only Gα_15_.

Thus, the classification of GPCRs according to their primary G protein coupling obtained through the relative ranking of all possible substrates generates significant insights filling information gaps and correcting existing knowledge.

### Rank ordering the G protein preferences of GPCRs establishes patterns and rules of engagement

Our dataset revealed that as many as 73% of tested GPCRs can activate multiple Gα across different classes (Figure 2D), indicating that the majority of GPCRs have secondary coupling. However, with traditional indirect methods, determining these secondary couplings with accuracy has been a challenge, as evident from the 27% agreement of our data with GtoPdb (Figure 2A).

To reveal the rules in the G protein-coupling preferences of GPCRs, we took advantage of the precise quantitative data that our approach generates and carefully analyzed the entire G protein couplings within our dataset (Figure 2E). In particular, we paid special attention to the rank order between individual Gα subfamilies coupling to a given GPCR. Overall, Gα_i/o_ was the most common substrate coupling to the vast majority of GPCRs, followed by Gα_15_ (Figure 2F), suggesting that it may be structurally hard for the GPCRs to avoid Gα_i/o_ and Gα_15_ coupling. On the other hand, the activation of Gα_s_, Gα_q_, and Gα_12/13_ is less common. In particular, as many as 78% of GPCRs were found not to be coupled to Gα_12/13_, suggesting specific constraints for the activation of these G proteins.

Interestingly, the rank order of the secondary couplings also followed a specific pattern (Figure 2G). Gα_15_ was the second-best universal substrate for Gα_i/o_-, Gα_q_-, and Gα_s_-coupled receptors. For the receptors that primarily couple to Gα_i/o_, secondary coupling dropped precipitously from Gα_15_ to ~6% for Gα_q_ and Gα_12/13_. Strikingly, Gα_i/o_-coupled receptors never activated Gα_s_, at least in the repertoire of GPCRs we have studied, suggesting that there is a mechanism that excludes Gα_s_ from being activated by Gα_i/o_-coupled receptors. Gα_q_-coupled receptors were markedly more promiscuous: nearly 80% of them readily activated Gα_15_ and Gα_i/o_, and many coupled to Gα_12/13_ and a few to Gα_s_. A similar pattern was observed for Gα_s_-coupled receptors with robust coupling to Gα_15_ followed by Gα_i/o_, Gα_12/13_, and Gα_q_.

Careful quantitative binning also allowed us to obtain several significant insights into the relationships between primary and secondary couplings while revising several dogmas (Figure 2G). First, we found that the coupling of GPCRs to both Gα_q_ and Gα_s_ is disfavored. Only 7% of the Gα_q_-coupled receptors activated Gα_s_. Conversely, only 21% of the Gα_s_-coupled receptors could activate Gα_q_. Therefore, there seems to be a mechanism that prevents Gα_q_ and Gα_s_ from being activated by the same receptors. Second, we found that Gα_s_ and Gα_q_ play a major role in the primary coupling, whereas Gα_15_ and Gα_12/13_ are mainly involved in secondary coupling (Figure 2E). In addition, Gα_i/o_ appears to contribute equally to both primary and secondary coupling.

### Reclassification of group I mGluRs

Analyzing the rank order of G protein selectivity revealed an unexpected result. We noted a strong preference for Gα_i/o_ in the majority of class C GPCRs (Figure 2C). In particular, metabotropic glutamate receptor 5 (mGluR_5_) (*GRM5*), which is historically classified as a Gα_q_-coupled receptor, showed the strongest activity on Gα_o_, making it essentially a Gα_i/o_-coupled receptor with secondary coupling to Gα_q_ and Gα_15_ (Figure 1I). Furthermore, we also detected a very strong Gα_i/o_ activity on mGluR1, which was comparable to Gα_q_, although this receptor still activated Gα_q_ statistically better than Gα_o_ (Figure 1I).

Because G_i/o_ coupling of group I mGluRs is not generally considered in the biological context, we next confirmed its physiological relevance in native neurons (Figure 2H). Using cultured striatal neurons that endogenously express mGluR5,^32,33^ we indeed observed that stimulation of mGluR5 with a specific agonist (CHPG) inhibits cAMP production in a PTX-sensitive manner (Figure 2I), and this effect can be inhibited by a mGluR_5_-specific antagonist (MTEP) (Figure 2J). These results demonstrate that mGluR_5_ indeed couples to Gα_i/o_ to propagate cellular response in the endogenous system. We further confirmed the G_i/o_ coupling of mGluR_5_ with an orthogonal assay (Figures S2C and S2D).

### GPCR classification based on rank order of G protein preferences

On the basis of our findings, we propose a taxonomy for the classification of GPCRs that takes into consideration not only primary and secondary but also the rank order of G protein preferences. Given the unique coupling property of Gα_15_ observed in this study, we propose to treat it separately for functional classification. Accordingly, GPCRs are classified based on the rank order of preferences for Gα subunits across five groups: Gα_i/o_, Gα_q_, Gα_15_, Gα_s_, and Gα_12/13_. For example, ETBR can be classified as a Gα_i/o_>Gα_q_,Gα_12/13_>Gα_15_-coupled receptor, separating significant differences by “>“ and insignificant ones by commas. Therefore, our quantitative kinetic measurements have revealed many previously undiscovered rules and patterns governing the G protein selectivity of GPCRs and have formed a basis for the functional classification of GPCRs.

### Machine learning predicts G protein preferences of GPCRs

**Figure 3.**
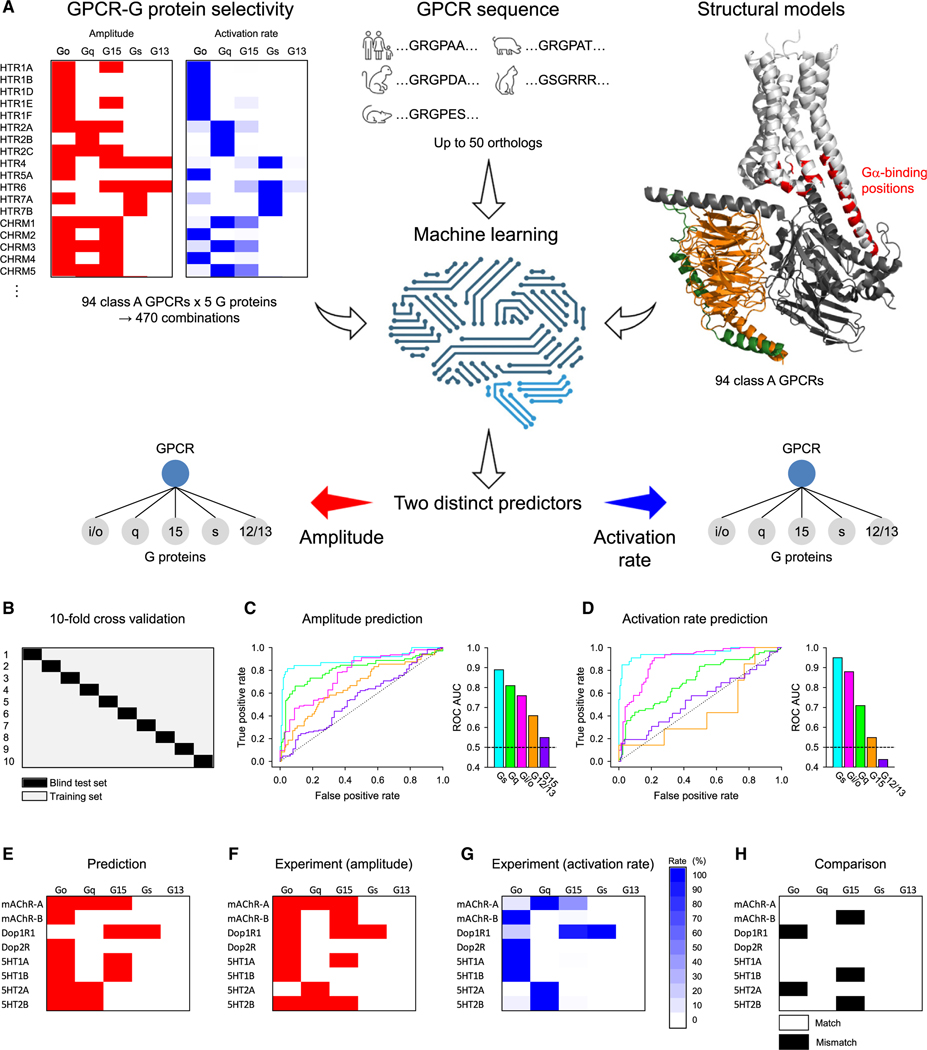
Unsupervised machine learning of predicting G protein coupling *(*A) Conceptual representation of the machine learning task. For training and constructing predictors based on amplitude and activation rate, we used the G protein selectivity, amino acid sequences of 50 orthologs, and structural models of 94 class A GPCRs examined in this study. (B) The framework of 10-fold cross-validation to develop machine learning models to predict the G protein selectivity of class A GPCRs. (C and D) ROC AUC values for the amplitude (C) and activation (D) predictors per G protein. (E) Deep learning model predictions of G protein selectivity of *Drosophila* GPCRs. (F and G) Experimental data of G protein coupling of the *Drosophila* GPCRs. (H) Comparison of prediction and actual results. Three independent experiments were performed, and the mean and SEM values are provided in Table S1.

Encouraged by quantitative kinetic information that discriminates ranking of G protein preferences for over a hundred GPCRs, we developed a set of machine learning-based predictors of class A GPCR-G protein couplings. We approached this by splitting the task into two binary classification problems: predicting whether a GPCR is coupled in principle to a G protein (amplitude >0%) and whether coupling leads to fast activation (activation rate >30%) (Figure 3A). Amplitude-based predictors cover entire coupling profiles, while activation-rate-based predictors narrow down to their preferential G proteins.

We focused specifically on class A GPCRs and their amino acid residues in the interface with the Gα subunit, according to the D_1_R/G_s_ complex (PDB: 7jvq) as a reference structure. We first aligned structural models of 94 class A GPCRs, which were functionally examined in this study, to the D_1_R model and mapped corresponding residues to the reference. In order to increase the power of the relatively limited training dataset, we included up to 50 ortholog sequences for each of the GPCRs, assuming the evolutionary conservation of sequences involved in determining G protein selectivity. We further deployed a sequence “embedding” protocol^34,35^ that uses unsupervised deep learning models to describe the properties of each residue in its unique environment and used a series of neural networks as binary classifiers to extrapolate this information on query interfaces.

To evaluate the performance of our predictors, we performed 10-fold cross-validation (Figure 3B) and obtained the area under the receiver operating characteristic curve (ROC AUC) (Figures 3C, 3D, and S3), which measures the performance of a binary classifier. For the metric, a value of 0.5 represents a random classifier, while a value of 1.0 reflects a perfect classifier. For amplitude prediction, our method achieves a micro-averaged ROC AUC of 0.85 and for activation rate, a micro-averaged ROC AUC of 0.85 (Figure S3A), indicating the high performance of our two predictors.

Evaluating each G protein class separately (Figures 3C, 3D, S3B, and S3C), we found that for both amplitude and activation rate, the prediction was the best for the G_s_ class (ROC AUCs of 0.89 and 0.95, respectively) followed by Gα_q_ and Gα_i/o_ with ROC AUCs 0.81 and 0.76, respectively, for amplitude prediction and 0.71 and 0.88 for activation rate prediction. In contrast, we observed no significant learning in either amplitude or activation rate for the Gα_12/13_ or Gα_15_ classes. These results suggest the existence of clearly discernable rules that govern the selectivity of GPCRs coupling to Gα_i/o_, Gα_q_, and Gα_s_ based on our characterization of kinetic properties of class A GPCRs.

Finally, to confirm the accuracy of our prediction algorithm, we predicted the G protein selectivity of acetylcholine, dopamine, and serotonin receptors from *Drosophila* and performed experiments in parallel (Figures 3E–3H). Since ROC AUC was better when predicted by amplitude (Figures 3C and 3D), the predictor established by amplitude was used. As a result, the prediction of G_15_ coupling, which had the lowest ROC AUC, failed in three cases (Figure 3H). The prediction and experimental results differed for Dop1R1 and 5HT2A for secondary G_o_ coupling. However, the prediction was successful, with a probability of 87.5%, arguing that even evolutionarily distant GPCRs can be predicted.

### Deciphering structural determinants of Gα selectivity of class A GPCRs

Our experimental analysis of the G protein-coupling patterns of GPCRs and the successful *in silico* prediction of GPCR-G protein selectivities prompted the investigation of the structural underpinnings underlying the selectivity. To tackle this unsolved crucial issue, we took advantage of many available structures of GPCR/G protein complexes^36,37^ and analyzed all 33 structures of class A receptors available while conducting this research (Table S2). We did not include class B and C GPCRs for their differences in residue-residue networks involved in the GPCR activation^38^ and distinct Gα-binding mechanisms.^39–42^

**Figure 4.**
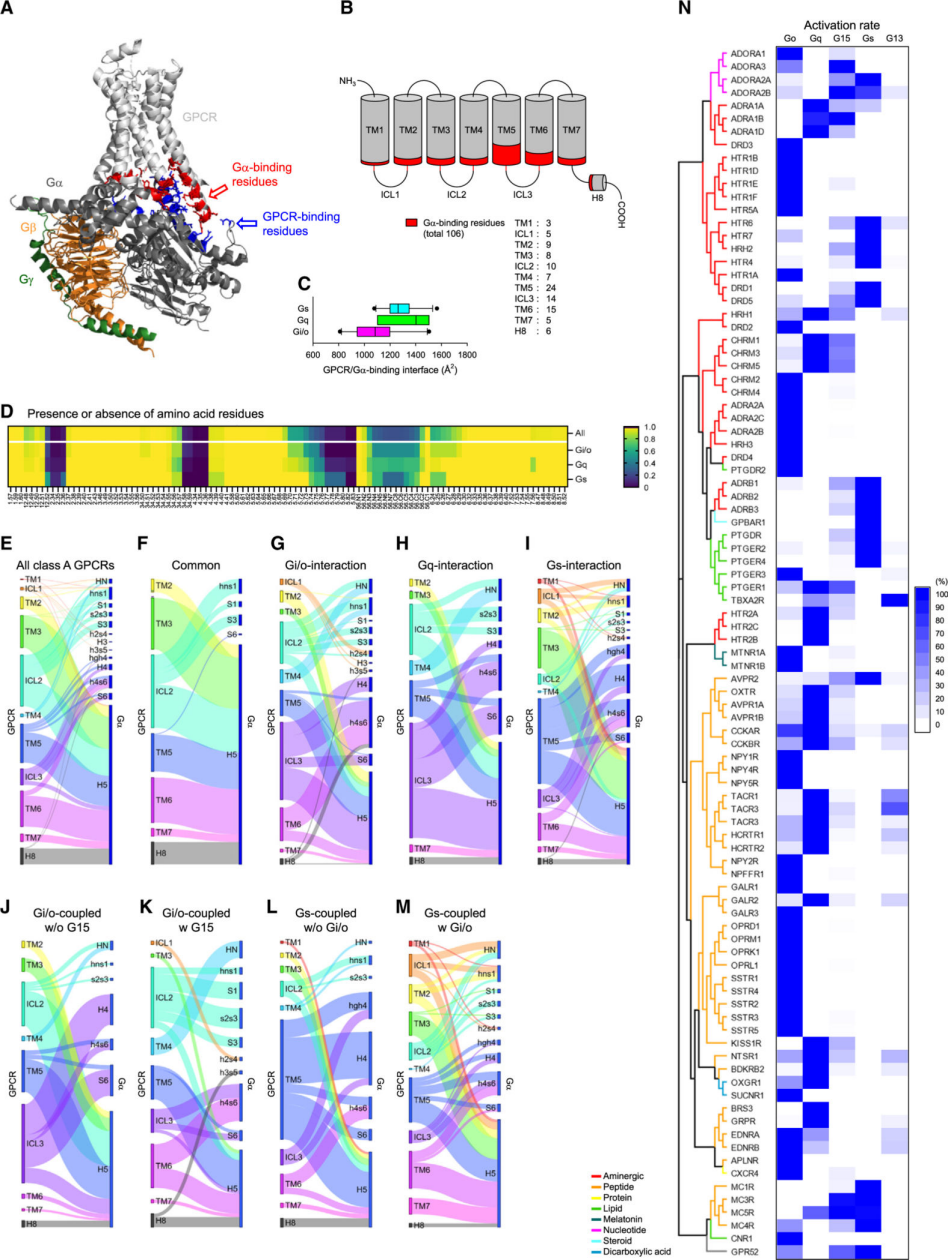
Gα-binding mechanism of class A GPCRs (A) Identification of the residue-residue contacts underling GPCR-G protein interaction. (B) The amino acids that bind Gα in the TMs and ICLs (Gα-binding residues) are indicated by red. (C) The area of interface involved in binding of GPCR and Gα. (D) The presence and absence of amino acid residues in the Gα-binding residues in class A GPCRs. (E–I) The interaction networks between structural elements in GPCR and Gα: all class A GPCRs (E), common to all interactions between class A GPCRs and Gα subunits (F), and specific for G_i/o_ (G), G_q_ (H), and G_s_ interaction (I). The width of the lines indicates the number of non-covalent contacts. The nodes represent the total number of residue-residue contacts for each structural element. (J and K) The interaction networks of Gi/o-coupled GPCRs that couple with only G_i/o_ (J) or both G_i/o_ and G_15_ (K). (L and M) The interaction networks of G_s_-coupled GPCRs without (L) or with (M) G_i/o_ coupling. (N) Relationship between Gα selectivity and amino acid sequence of Gα-binding residues.

We hypothesized that Gα selectivity is predominantly determined by the Gα-binding surface of GPCRs. To test this, we first identified the amino acid residues within 5 Å from each other at the GPCR-Gα interaction interface (Figure 4A; Table S2). The amino acid residues found in this analysis encompassed all structural elements of GPCR facing cytoplasm, including seven transmembrane helices (TM), three intracellular loops (ICL), and C-terminal α-helix 8 (H8) containing 106 individual amino acid positions in GPCR (Figure 4B) and 74 amino acid positions in Gα (Figure S4). These residues were differentially distributed to accommodate differences in the Gα-binding surface across GPCRs, which varied from 811 to 1565 Å depending on the structure and the identity of Gα bound (Figure 4C). These differences in the size of the Gα-binding pocket were largely driven by the smaller number of amino acids in TM5 and ICL3 in Gα_i/o_-coupled receptors (Figure 4D), possibly contributing to Gα selectivity of these receptors.

In order to probe the contributions of these elements to G protein selectivity, we scrutinized the residue-residue networks between GPCRs and Gα (Figures 4E–4I). We relied on the common Gα numbering system in GProteindb (Figure S4)^43^ and the common GPCR numbering system^44^ for residue attributions while modifying the numbering of the three intracellular loops of GPCRs (Table S3). Using these numbering systems, we built and integrated an interaction network map of all GPCRs-Gα contacts (Figure 4E). This analysis indicates that all the structural elements of GPCR facing the cytoplasm are used for binding to Gα, although the degree of involvement is different. Conversely, the Gα proteins also rely on as many as 13 structural elements for GPCR binding, with the C-terminal α-helix 5 (H5) responsible for approximately half of the interactions.

From this integrated map, we next extracted a network common across all GPCRs and G proteins (Figure 4F) as well as networks that are specific to individual Gα types (Figures 4G–4I). Interestingly, for common interactions with the Gα, GPCRs utilized ICL2 and H8, as well as TM domains other than TM1 and TM4 (Figure 4F). All of these structural elements except ICL2 are bound mainly to H5, but ICL2 much broadly interacted with hns1, S1, and S3 as well as to H5 in Gα. In contrast, the contacts with individual Gα types showed a wider range of elements engaged on both GPCRs and Gα sides (Figures 4G–4I). Interestingly, interactions with Gα_i/o_ and Gα_q_ followed the same pattern, with the major difference being that GPCRs heavily used TM6 for Gi/o interaction but not for the interaction with Gα_q_. Interestingly, coupling to Gα_s_ involved a more complex mechanism. A unique feature of Gα_s_ coupling is a significantly higher reliance on TM3 and TM5 amid the scarce contribution of ICL2 and ICL3 of GPCRs for interaction. The most distinctive feature of GPCR-Gα_s_ interaction is the binding of Gα_s_ to TM5 and ICL3 of GPCRs through a long hgh4 loop that is not found in other Gα. These observations suggest that multiple structural elements are differentially engaged in determining the selectivity of GPCR-Gα recognition.

In order to determine how identified elements shape the secondary G protein coupling selectivity, we further considered the rank order of G protein selectivity. First, we compared the residue-residue networks for Gα_i/o_-coupled receptors that can or cannot couple to Gα_15_ (Figures 4J and 4K). The receptors with the ability to couple to Gα_15_ abrogated contacts between ICL3 and H4, weakened ICL2-H5 interaction, and strengthened TM4-HN and ICL2-s2s3 contacts. Remodeling of ICL2 interactions is perhaps the most prominent determinant defining the difference between these two types of GPCRs.

Similar remodeling of the interaction network was also evident for G_s_-coupled receptors that allow or do not allow additional Gα_i/o_ coupling (Figures 4L and 4M). Here, marked differences in the involvement of ICL1 and TM5 were notable. Although more structures need to be elucidated for a more detailed analysis on the secondary coupling, these analyses strongly suggest that the mode of primary coupling is significantly influenced by their secondary coupling.

To see whether different GPCRs utilize the same set of amino acids in their Gα-binding regions to discriminate between various Gα, we conducted a phylogenetic analysis of the Gα binding residues from all class A GPCRs functionally examined in this study (Figure 4N). The resultant phylogenic tree revealed that individual receptors clustered together based on their type of ligand to be bound rather than the similarity of their G protein coupling profiles. This result suggests that the selectivity determinants for G proteins in GPCRs may have convergently evolved independently from the elements necessary for ligand binding and the structural changes associated with activation.^45^ As such, we concluded that the Gα selectivity of GPCRs depends on the similar three-dimensional structures created by different arrangements of amino acid sequences.

### Engineering GPCRs to alter their G protein selectivity

**Figure 5.**
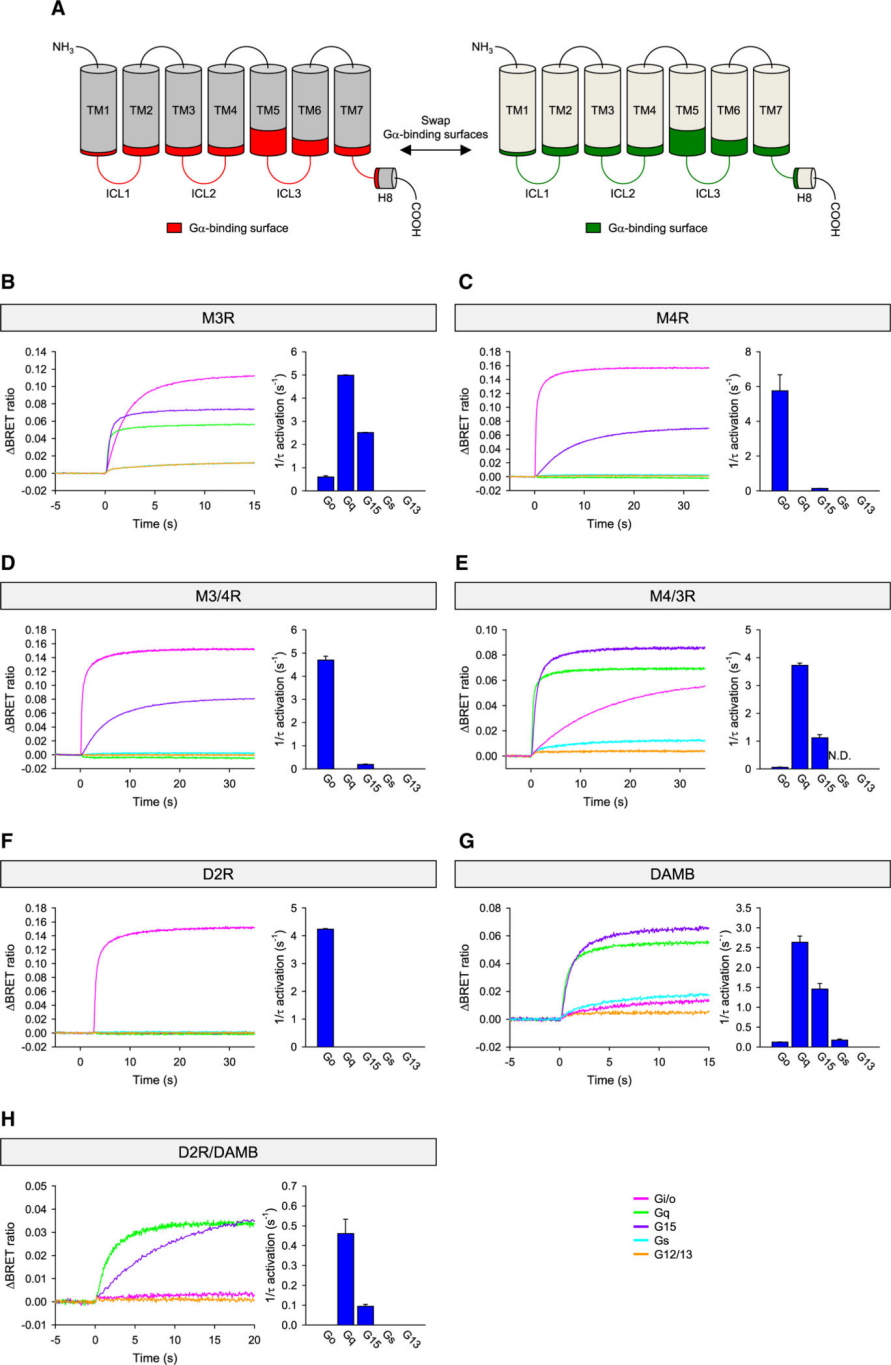
Experimental validation of GPCR-Gα selectivity determinants (A) The Gα-binding surface and the strategy of making chimeras. (B–E) The G protein-coupling profiles of wild-type M_3_R and M_4_R, and M_3/4_R and M_4/3_R chimera. M_3/4_R is the chimera with a transplantation of the Gα-binding surface of M_4_R into the backbone of M_3_R, while M_4/3_R is the opposite. (F–H) G protein-coupling profiles of wild-type D_2_R and DAMB and D_2_R/DAMB chimera. The Gα-binding surface of DAMB was transplanted into D_2_R. G protein activation rate constants were plotted as bar graphs (B–H). A minimum of three independent experiments were performed, and the mean and SEM are shown in the bar graphs. N.D., not determined.

Identification of the determinants in the GPCRs involved in selective Gα recognition prompted us to address their sufficiency in dictating the G protein coupling properties of GPCRs. Since amino acids of GPCRs involved in Gα selectivity are widely distributed with limited coordination of their positions, we adopted a swapping transplantation approach where the three loops were included as part of the “Gα-binding surface” (Figure 5A).

For the initial experiments, we chose muscarinic acetylcholine receptors ((M_3_R) (*CHRM3*) and (M_4_R) (*CHRM4*)). According to our analysis, M_3_R is a Gα_q_>Gα_15_>Gα_i/o_-coupled receptor (Figure 5B), whereas M_4_R is a Gα_i/o_>Gα_15_-coupled GPCR (Figure 5C). Transplanting the Gα-binding surface from M_4_R onto M_3_R resulted in the perfect conversion of the G protein-coupling profile of M_3_R to that of M_4_R (Figure 5D). Conversely, replacing the Gα-binding site of M_4_R with that of M_3_R also transformed its coupling profile to Gα_q_>Gα_15_>Gα_o_, a nearly identical pattern seen for parental M_3_R (Figure 5E).

In the next set of experiments, we chose less related GPCRs by increasing the evolutionary distance between the pair. For this, we used the human D2 dopamine receptor (D_2_R) (*DRD2*), which is strictly Gα_i/o_ selective (Figure 5F), and the *Drosophila* dopamine receptor (DAMB) (*Dop1R2*), which has an unusually ubiquitous profile Gα_q_>Gα_15_>Gα_s_,Gα_o_ (Figure 5G).^46^ Strikingly, replacing the Gα-binding surface in D2R for that from DAMB converted its primary selectivity to the intended Gα_q_>Gα_15_ (Figure 5H). However, the chimeric receptor was unable to couple to either Gα_s_ or Gα_o_, presumably because of its low expression level, which is evident from the slow activation rates from Gα_q_ and Gα_15_. Thus, we conclude that major G protein selectivity determinants of GPCRs are indeed contained within the Gα-binding surface that we defined based on our structural analysis, even in evolutionarily distant GPCRs.

### Natural genetic variants in the Gα-binding residues impact the Gα selectivity of GPCRs

Knowing the functionally important sites of a protein at the amino acid level has direct implications for predicting the impact of mutations on protein function. Recently, there has been an explosion of genomic information identifying many missense variations (MVs) in GPCRs associated with diseases^7^ and non-disease traits.^47^ However, the functional implications of these polymorphisms are far from being completely understood.

**Figure 6.**
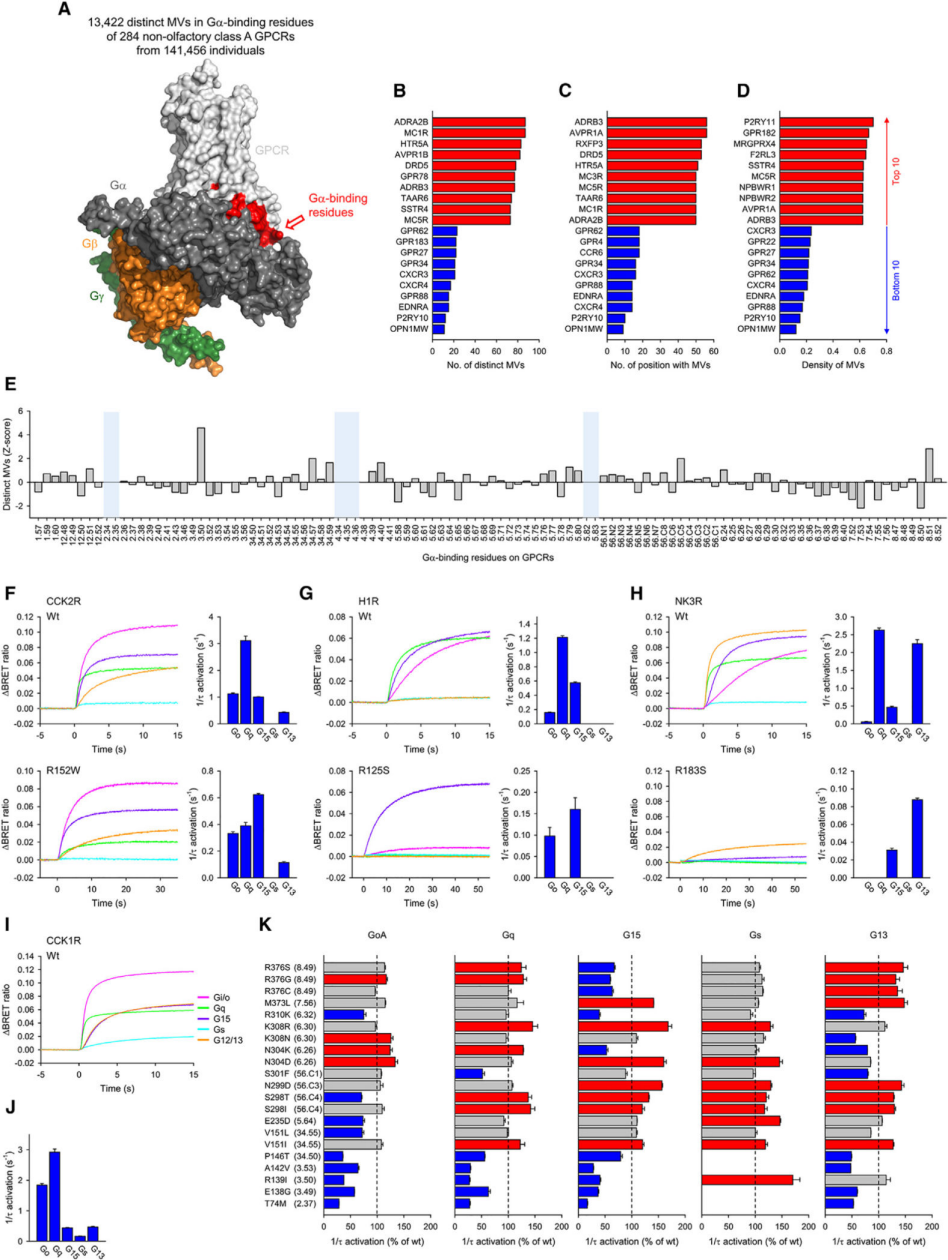
Impact of natural genetic variations on Gα selectivity (A) The number of unique MVs in Gα-binding residues. (B–D) The ranking of the number of distinct MVs in the Gα-binding residues. (C) The ranking of the number of positions containing MVs in the Gα-binding residues. (D) The density of MVs in the Gα-binding residues. The density of MVs as calculated by the number of MVs divided by the number of amino acid residues in Gα-binding residues of each GPCR. (E) *Z* score of MVs. The regions (notated in light blue) with fewer than 19 GPCRs were not used in the calculation of the *Z* score. (F–H) Characterization of the 3.50 mutants of CCK_2_R, H_1_R, and NK_3_R. (I and J) The response of wild-type CCK_1_R to 1 μM CCK-8. Time course (I) and activation rates (J) of CCK_1_R-induced G protein activation are shown. (K) Impact of mutations on activation rates. A minimum of three independent experiments were performed, and the mean and SEM are shown in the bar graphs. The traces are the average values from three independent experiments.

Leveraging our analysis of structural determinants underlying G protein selectivity of GPCRs, we first surveyed natural genomic variations in the human population. For 284 non-olfactory class A GPCRs in the genome aggregation database (gnomAD) from 141,456 human subjects,^48^ we found 13,422 unique MVs with a mean of 50 rare (frequency <2%) and 0.1 common (frequency >2%) variants per GPCR (Figures 6A and S5A; Table S4). Remarkably, every position except 4.34 and 5.83 contained at least one MV (Figure S5B). Considering the number of cohorts and the number of unique mutations together with the frequency of mutations, 99.88% of people have some mutations in their Gα-binding residue (Figure S5C), indicating the importance of characterizing their impact on function.

We next looked at individual GPCRs and determined the number of different mutations (Figure 6B), the number of sites where the mutations were introduced (Figure 6C), and the densities of mutations (Figure 6D). The highest number of missense mutants were observed in the α_2B_ adrenergic receptor (α_2B_AR) (*ADRA2B*) (Figure 6B), suggesting that this receptor is actively undergoing changes in its G protein recognition properties. The β_3_-adrenergic receptor (*ADRB3*) and vasopressin 1A receptor (*AVPR1A*) were also the most widely mutated (Figure 6C), which may reflect greater heterogeneity in their Gα selectivity. The highest mutation burden was found in P2Y purinoceptor 11 (*P2RY11*) (Figure 6D), in which ~70% of the Gα-binding residues were polymorphic. On the other side of the spectrum, we found several receptors with very low mutation incidence, including P2Y purinoceptor 10 (P_2_Y_10_) (*P2RY10*), chemokine receptor CXCR_3_ (*CXCR3*), chemokine receptor CXCR_4_ (*CXCR4*), G protein-coupled receptor 183 (*GPR183*), and G protein-coupled receptor 34 (*GPR34*) (Figures 6B–6D).

The identification of a large number of mutations in the Gα-binding residues of GPCRs begs the question about their functional relevance. Applying *Z* score analysis to determine the frequency of MVs variation for each position (Figure 6E) revealed that two positions, 8.50 and 7.53, were clear outliers (Figure S5E) with the lowest scores suggesting the conservation of these amino acid residues making their alterations incompatible with general functional activities of GPCRs.

Unexpectedly, we found many mutations in conserved positions well known for their functional importance. For example, positions 3.50 and 8.51 had a large number of mutations. The arginine residue in the 3.50 position is particularly well conserved among GPCRs and is a part of the functionally important DRY motif that directly binds to the C terminus of Gα (Table S2).^49^ The MVs in this residue are heterozygous and rare with <1% minor allele frequency.

To test implications of the variations, we performed functional analysis of missense mutants found in 3.50 in several GPCRs: D_5_R (*DRD5*), V_2_R (*AVPR2*), H_1_R (*HRH1*), OX_1_R (*HCRTR1*), NK_2_R (*TACR2*), NK_3_R (*TACR3*), CCK_2_R (*CCKBR*), and B_2_R (*BDKRB2*). As expected, mutating this residue was not tolerated, with a vast majority of receptors completely losing their function (Figures S6A–S6E). However, variants in three receptors retained a readily measurable activity (Figures 6F–6H) and showed substantial alteration in the rank order of G proteins coupling specificity. Thus, 3.50 residue, in addition to being important for the interactions with Gα in general, also specifies their selectivity.

To understand the broader implications of non-synonymous variants across different positions in GPCRs, we studied the CCK_1_R receptor, which can activate nearly all types of G proteins.^47^ A total of 57 missense mutations were found within the Gα-binding residues in CCK_1_R, and 21 of those were functionally tested (Figures 6I–6K, S6F, and S6G). All mutants affected the coupling to at least one of the five G proteins we tested (Figure 6K). Three of these mutants, in particular, were found to affect their primary coupling (Figure S6G). In some cases, exemplified by the R139I mutant, the preference of the receptor for Gα_q_ vanished, and it activated its secondary substrate Gα_i/o_ equally well. In other cases, e.g., A142V and S301F mutants, the selectivity was reversed for the receptors to prefer Gα_i/o_ as a primary substrate. Overall, these results suggest that most amino acid alterations in Gα-binding residues of GPCRs alter their Gα selectivity with likely physiological implications.

## DISCUSSION

Identifying which G proteins are activated by GPCRs is essential for understanding their physiological functions.^12,50^ In this study, we define G protein coupling profiles of over a hundred of non-sensory GPCRs across three main classes. We observed that the majority of GPCRs that we studied coupled to multiple G protein subfamilies with varying efficiency and kinetics. Thus, GPCRs are endowed with the capacity to initiate waves of multimodal signals. We think that this coupling diversity is the essential property of GPCRs, allowing them to control a broad range of physiological functions underpinning their evolutionary success. Thus, the currently used functional classification of GPCR according to their primary G protein coupling may inadequately describe their actions. Instead, we propose a more fine-grained, rank-order-based GPCR classification based on quantitative analysis of their temporal G protein coupling diversity to capture their physiological functions better.

### Non-invasive approach to unveiling G protein coupling selectivity of GPCRs

Understanding the G protein coupling selectivity of GPCRs has been a major goal in the field since their discovery.^12^ Traditionally, these investigations have relied on downstream signaling pathways as readouts,^51,52^ which complicates the interpretations due to the inability to distinguish between individual G proteins with similar activities, signaling crosstalk, and the need to employ multiple experimental systems with different sensitivities.

The advent of biosensors circumvented this issue and provided the opportunity to monitor signaling events directly.^53–55^ However, these approaches are faced with limitations that stem from the need to modify Gα and/or GPCRs. In contrast, our study employs a completely different strategy for monitoring G protein activation by GPCRs that does not require any modification of GPCRs and Gα whose interaction is being studied. We monitor the interaction of the Gβγ and effector molecules, which is a common denominator in the activation of all G proteins. This approach allows straightforward comparison and quantification of relative Gα engagement by a given GPCR in a single platform.

### Deciphering the rules and patterns of GPCR-G protein coupling: Insights into selectivity and functional diversity

Our analysis of G protein selectivities of GPCRs revealed previously unknown patterns offering significant insights into possible rules that govern GPCR-G protein recognition. Overall, we found the vast majority of GPCRs (~73%) to be promiscuous, suggesting the physiological importance of multi-G protein coupling across G protein subfamilies. The degree of promiscuity varied across groups of GPCRs. For instance, more than half of the GPCRs with primary Gα_i/o_ coupling only couple to Gα_i/o_, and none of them activated all five G proteins. On the other hand, about 90% of the Gα_q_-and Gα_s_-coupled receptors were found to be promiscuous. These observations are consistent with the larger size of the Gα-binding pocket in Gα_s_- and Gα_q_-coupled receptors. Thus, the degree of promiscuity may be determined, at least in part, by structural constraints to accommodate specific types of Gα.

Interestingly, the secondary coupling of GPCRs follows certain patterns. While GPCRs mainly use Gα_s_ and Gα_q_ as primary coupling partners, Gα_i/o_ is engaged as both primary and secondary partners, suggesting the importance of Gα_i/o_ signaling not only as a primary modality but also as a modifier of Gα_q_- and Gα_s_-coupled receptors. We have also revealed the existence of receptors that are primarily coupled to Gα_12/13_ and Gα_15_. These examples are, however, exceedingly rare, as most GPCRs use Gα_12/13_ or Gα_15_ as secondary coupling partners, suggesting that the cellular signaling logic dictates that these G proteins may need to work in concert with other G proteins. Nevertheless, we find GPCRs that do not couple to Gα_15_, indicating that there are situations where activating this G protein needs to be avoided. Although there are notable exceptions, another rather common pattern is mutual exclusivity in the activation of Gα_q_ and Gα_s_.

Despite generalization of patterns gleaned from the behavior of many GPCRs, there are also outliers that break these rules, suggesting that any G protein coupling combinations are possible in principle.

### Implications from machine-learning-based predictions

Despite the complexity in observed patterns of G protein selectivity and high promiscuity of GPCRs, we were able to develop a machine learning algorithm that could predict the G protein preferences of GPCRs. Its success rate was the highest for Gα_s_-coupled receptors with moderate success for G_q_- and G_i/o_-coupled receptors. However, it showed poor performance in predicting Gα_12/13_ and Gα_15_ coupling. While its limited success with Gα_12/13_ could be explained by the small number of examples of GPCRs activating these G proteins in our dataset, poor performance on Gα_15_ despite the ample amount of Gα_15_ activation by GPCRs suggests that there is no conserved mechanism for GPCR-Gα_15_ coupling. The latter might be the explanation for the ubiquitous Gα_15_ coupling to many GPCRs.^30^ The absence of a rule also appears to involve the Gα_15_ coupling of all of the designer GPCRs, suggesting the difficulty of avoiding Gα_15_ coupling by artificially designed GPCRs.

### Integrating structural analysis for functional manipulation of GPCRs

Our analysis yielded significant insights regarding the GPCR-G protein coupling mechanism, revealing a higher level of complexity than previously believed. Contrary to the notion that simple determinants of G protein selectivity exist,^56^ our study revealed intricate interconnections between various structural elements of GPCRs and Gα subunits. Despite extensive experimental investigations with chimeric GPCRs^57^ and comprehensive big data analyses,^45,58^ no definitive rules governing G protein selectivity have been identified, impeding our ability to manipulate it.

Our study demonstrates that G protein selectivity can be modified by altering GPCR sequences based on theoretical predictions derived from structural analysis. By exchanging the G protein-binding surface, we are able to alter not only primary but also secondary coupling of G proteins. The identification of the selectivity determinants was made possible by combining comprehensive structural analysis of all GPCR/G protein complexes with an exhaustive functional dataset acquired using the principles developed in this work. This contrasts with a traditional sequence homology-based approach^45^ or analyzing the structures of individual complexes^36^ attempted before.

The goal of earlier studies has been to identify the minimum unit involved in dictating the G protein selectivity. However, attempts to alter the selectivity by designing chimeric GPCRs swapping limited sequence elements have not yielded satisfying outcomes.^56^ Our study provides an explanation for this difficulty by showing that structural elements involved in discriminating G proteins are distributed even broader than appreciated before.^45^ Thus, leveraging advances in structural analysis and the functional evaluation strategies employed in our study now allows obtaining further insights into the intricacies of GPCR-G protein selectivity. This knowledge can be utilized for protein engineering, enabling design of GPCRs with new functionalities, as well as for understanding how natural genetic variations may underpin signaling deficiencies.

### Limitations of the study

Our experimental system measures the most upstream signaling event that is likely minimally affected by variations in cell types. However, the reported data may not exhaustively capture all possible coupling modalities that could be realized in specialized native cells, a limitation common to all studies using reconstituted systems.

This study examined the rank order of G protein coupling selectivity of GPCRs by selecting five representative G proteins. However, the human genome encodes 16 Gα subunits with distinct biochemical properties. Subsequent studies could extend our findings by expanding all 16 Gα members.

The physiological significance of the promiscuous G protein coupling profiles that we observe for many GPCRs remains un-defined. Nevertheless, the discovery of previously unknown rank orders of G protein selectivity reported in this manuscript is expected to make a significant contribution to future research in elucidating the physiological functions of GPCRs and in drug discovery.

## STAR★METHODS

### RESOURCE AVAILABILITY

#### Lead contact

Further information and requests for resources and reagents should be directed to and will be fulfilled by the lead contact, Kirill Martemyanov (kmartemyanov@ufl.edu).

#### Materials availability

Plasmids generated in this study will be made available upon request.

#### Data and code availability

The raw data derived from the BRET assay has been documented in Table S1. This reported original code and available at https://zenodo.org/record/8271720 (https://doi.org/10.5281/zenodo.8271720). Any additional information required to reanalyze the data reported in this work paper is available from the lead contact upon request.

### METHOD DETAILS

Precise details of all the procedures in the paper were provided in STAR Methods.

#### cDNA constructs

Gα_oA_ (NM_020988), Gα_15_ (AF493904), and Gα_13_ (NM_006572) in pcDNA3.1(+) were purchased from cDNA Resource Center (www.cDNA.org). The pCMV5 plasmids encoding human Gα_q_ and bovine Gα_s_ short isoform were gifts from Dr. Hiroshi Itoh. Venus 156–239-Gβ_1_ (amino acids 156–239 of Venus fused to a GGSGGΓ linker at the N terminus of Gβ_1_ without the first methionine (NM_002074)) and Venus 1–155-Gγ_2_ (amino acids 1–155 of Venus fused to a GGSGGΓ linker at the N terminus of Gγ_2_ (NM_053064)) were gifts from Dr. Nevin A. Lambert.^24^ Flag-tagged Ric-8A (NM_053194) in pcDNA3.1 was a gift from Dr. Jean-Pierre Montmayeur.^60^ Flag-tagged Ric-8B (NM_183172 with one missense mutation (A1586G)) in pcDNA3.1 was a gift from Dr. Bettina Malnic (Von Dannecker et al., 2006). The masGRK3ct-Nluc-HA constructs were constructed by introducing HA tag at the C terminus of masGRK3ct-Nluc reported previously.^15^ PTX-S1 constructs were reported previously.^61^ The GPCRs used in this study was listed in Table S1. GenBank accession number for each sequence is given in parentheses. DORA-RhoA (BRET), CalfluxVTX,^62^ Gα_oA_-Nluc,^27^ and p115RH-AU5-CAAX in pcDNA3.1(+) were synthesized by GenScript. pENN.AAV.hSyn.Cre.WPRE.hGH was a gift from James M. Wilson (Addgene plasmid # 105553).

#### Transfection

HEK293T/17 cells were grown in DMEM supplemented with 10% FBS, minimum Eagle’s medium non-essential amino acids, 1 mM sodium pyruvate, and antibiotics (100 units/mL penicillin and 100 μg/mL streptomycin) at 37^◦^C in a humidified incubator containing 5% CO_2_. For transfection, cells were seeded into 3.5-cm dishes at a density of 2 × 10^6^ cells/dish. After 2 h, expression constructs (total 5 μg/dish) were transfected into the cells using PLUS (5 μL/dish) and Lipofectamine LTX (6 μL/dish) reagents. The Gα (Gα_oA_ (2), Gα_q_ (2), Gα_15_ (2), Gα_s_ short (6), or Gα_13_ (4)), Venus 156–239-Gβ_1_ (1), Venus 1–155-Gγ_2_ (1), masGRK3ct-Nluc-HA (1) were transfected. Gα_15_ was transfected with Ric-8A (1). A construct carrying catalytic subunit of pertussis toxin PTX-S1 (1) were transfected with Gα_q_, Gα_15_, Gα_s_, or Gα_13_ to inhibit the possible coupling of endogenous G_i/o_ to GPCRs. In order to monitor the dissociation of Gα_oA_ and Gβγ upon the activation of mGluR5, mGluR5 (2), Gα_oA_-Nluc (0.1), Venus 156–239-Gβ_1_ (1), and Venus 1–155-Gγ_2_ (1) with or without PTX-S1 (1) were transfected. To examine the G_12/13_ coupling of TBXA2R, TBX2AR (2) and DORA-RhoA (BRET) (1) with or without PTX-S1 (1) and p115RH-AU5-CAAX (1) were transfected. An empty vector (pcDNA3.1(+)) was used to normalize the amount of transfected DNA. The number in parentheses indicates the ratio of transfected DNA (ratio 1 = 0.21 μg).

#### *In cellulo* GEF assay

Cellular measurements of agonist-induced BRET responses between Venus-Gβγ and GRK3ct-Nluc-HA sensors were performed in living cells (described in detail in^15,63^). Sixteen to 24 h post-transfection, HEK293T/17 cells were washed once with BRET buffer (Dulbecco’s Phosphate-Buffered Saline (PBS) containing 0.5 mM MgCl_2_ and 0.1% glucose) and detached by gentle pipetting over the monolayer. Cells were harvested with centrifugation at 500*g* for 5 min and resuspended in BRET buffer. Approximately 50,000 to 100,000 cells per well were distributed in 96-well flat-bottomed white microplates (Greiner Bio-One). The Nluc substrate, furimazine, was purchased from PromeGα and used according to the manufacturer’s instruction. BRET measurements were made using a microplate reader (POLARstar OmeGα or PHERAstar *FSX*; BMG Labtech) equipped with two emission photomultiplier tubes, allowing us to detect two emissions simultaneously with the highest possible resolution of 20 ms per data point. All measurements were performed at room temperature. The BRET signal is determined by calculating the ration of the light emitted by the Venus-Gβγ (535 nm with a 30 nm band path width) over the light emitted by the Nluc (475 nm with a 30 nm band path width). The average baseline value (basal BRET ratio) recorded prior to agonist stimulation was subtracted from the experimental BRET signal values to obtain the resulting difference (ΔBRET ratio). The largest ΔBRET ratio was plotted as maximum BRET amplitude. The rate constants (1/τ) of the activation phase were obtained by fitting a single exponential curve to the traces with Clampfit 10.3.

#### Primary cultures of striatal neurons

The animal studies were carried out in accordance with the National Institutes of Health guidelines and were granted formal approval by the Institutional Animal Care and Use Committee of The Scripps Research Institute (approved protocol #16–032).Striatal neuronal culture was done as previously described.^59^ Briefly, striatum was dissected from homozygous CAMPER pups at P0 age in ice-cold HBSS supplemented with 20% FBS. Striatal tissue was washed twice in HBSS before digestion at 37^◦^C for 15 min in a buffer (pH 7.2) containing 137 mM NaCl, 5 mM KCl, 7 mM Na_2_HPO_4_, 25 mM HEPES, and 0.3 mg/mL papain. Then Striatal tissue was washed three times with HBSS (20% FBS), three times with HBSS, and three times with growth media (Neurobasal-A containing 2 mM GlutaMAX, 2% B27 Supplement serum-free, and 1% Penicillin-Streptomycin). Striatal tissue was then dissociated through pipetting 15 times with a standard P1000 pipette in the presence of DNase I (0.05 U/mL) and plated on poly-D-lysine coated glass coverslips. The cells were maintained in a humidified incubator at 37^◦^C and 5% CO_2_. Half of the growth media was changed every three days. For cAMP imaging, AAV-Cre, under the control of synapsin promoter, was added to the CAMPER striatal neuronal culture at DIV3 and incubated for 10–15 days. When required, striatal neuron culture was treated overnight with 1 μg/mL pertussis toxin (PTX, Tocris) before imaging. Since overnight incubation with 1 μg/mL PTX decreased the basal concentration of cAMP, 50 mM forskolin was used to elevate the basal cAMP concentration.

#### Live imaging of cAMP dynamics in primary medium spiny neurons

Primary neuronal cultures were imaged by a Leica TCS SP8 confocal microscope through a 253 objective lens. Briefly, excitation of mTurquoise FRET donor with a 442 nm laser was paired with simultaneous acquisition of XYZ image stacks at 10 s intervals. Fluorescence signal was collected through two HyD detectors tuned to 465–505 nm (mTurquoise FRET donor) and 525–600 nm (Venus FRET acceptor). FRET or donor/receptor ratio was calculated by using ImageJ to quantify the fluorescence intensity of the neuronal cell body. The FRET ratio was converted to the concentration of cAMP using a dose-response curve to cAMP standards in permeabilized neurons.^59^ Metabotropic glutamate receptor 5 (mGluR5) selective agonist CHPG (Tocris) and selective antagonist MTEP hydrochloride (Tocris) were bath applied to neurons during continuous perfusion at 2 mL/min in a pH 7.3 buffer consisting of 1.3 mM CaCl_2_, 0.5 mM MgCl_2_, 0.4 mM MgSO_4_, 0.4 mM KH_2_PO_4_, 4.2 mM NaHCO_3_, 138 mM NaCl, 0.3 mM Na_2_HPO_4_, 5.6 mM D-Glucose, and 20 mM HEPES.

#### Machine learning prediction of GPCR-G protein coupling

##### Defining a conserved reference interface for GPCRs

The structure of D1R/G_s_ complex (PDB: 7jvq) was used as a template. All D_1_R residues within 4Å of any residue of the Gα subunit were labeled as interface residues. A model or structure of each GPCR in its active state was obtained from either the GPCR database,^64^ RosettaFold models,^65^ or the Protein DataBank (https://www.rcsb.org/). Each model was then aligned to the structure of D_1_R and the closest residue after alignment to each interface residue in D_1_R (up to a maximum distance of 5Å ) was labeled as the corresponding interface residue.

##### Database of GPCR orthologs

A total of 94 Class A GPCRs were selected as the dataset. The sequence of each Class A GPCR was downloaded from UniPort (https://www.uniprot.org/ ),^66^ and then the corresponding cluster of sequences at 50% homology to the GPCR (Uniref. 50) was downloaded from Uniref (https://www.uniprot.org/uniref )^67^. Every sequence from the cluster was then aligned to the human protein, and corresponding interface residues were labeled according to those in the human structure model.

##### Embedding GPCR sequences using a protein language model

The pretrained protein BERT model from the ProtTrans suite^68^ was installed using the Transformers library (https://huggingface.co/docs/transformers/index). Briefly, ProtTrans exploited state-of-the-art tools from the natural language processing field, to train an auto-encoder model (BERT) on nearly 400 billion amino acids, producing a pretrained representation of protein sequences. ProtTrans has been shown to outperform existing methods across a wide variety of tasks. Using the prot_bert model, every residue from every GPCR sequence from the 94 uniref clusters was converted to a numerical representation of 1024 real values. This representation has been shown to embed structural and biophysical features of the residue in the context of its sequence (https://huggingface.co/docs/transformers/index) and to perform well in tasks with a small number of training examples.

##### Input to the machine learning predictor

The input to the neural network was a tensor of size Bx30×1024, the first dimension being the batch size (B = 32), the second dimension being the number of residues in the consensus interface (30), and the third dimension (1024) being the size of the pretrained sequence embedding for each amino acid residue. In cases where a residue resulted in a gap in the alignment, a value of 0 was assigned for the input.

##### Neural network architecture of the machine learning predictor

We divided the prediction problem into two binary classification tasks, activation kinetics and amplitude. In addition, each task could be devised as five binary classification problems, with the coupling to each G protein by itself a separate classification problem. Although this could be tackled with a multi-label neural network classificatory, our experiments showed no benefit from a single network and instead ran 5 neural networks for each task (one per G protein), for a total of 10 neural network classifiers. The neural network architecture consisted of two fully connected layers, (128 and 16 neurons, respectively), followed by a flattening layer, and three fully connected layers (128, 32, 4 neurons, respectively), and an output layer (1 neuron). Every inner layer was activated by a rectified linear unit (ReLU) function, and followed by batch normalization and dropout. The output layer was activated by a sigmoid function, and we used a binary cross entropy loss. The neural network was implemented in Keras (https://keras.io/).

##### Training and testing the machine learning predictor with 10-fold cross-validation

The dataset of 94 class A GPCRs was randomly divided into a training set (60% of the set), a validation set (20% of the set), and a testing set (20% of the set). Given the small size of the datasets, the exact distribution of training and testing sets can have a substantial effect on the performance of learning algorithms. Therefore, the learning procedure was repeated ten times with different random selections of testing and training sets (known as 10*-fold cross-validation*). Afterward, all testing sets were collated to compute performance metrics.

##### Metrics used to assess the performance of the machine learning predictor

The Area Under the Curve (AUC) of the Receiver Operating Characteristic (ROC) was used as the performance metrics. The ROC measures the performance of a binary classifier under different thresholds, and the ROC curve plots the behavior of the true positive rate vs. the false positive rate. The AUC of the ROC curve is a value between 0 and 1, with 0.5 being the AUC of a random predictor and 1.0 being the AUC of a perfect predictor. Since each of the two classification tasks contained a maximum of five labels (*e*.*g*., a binary predictor for each of the five G proteins), we used two metrics to measure the performance of the predictor across all classes: micro-averaged ROC AUC and macro-averaged ROC AUC. Briefly, the macro average is calculated by summing the ROC AUCs of all classes (in this case the ROC AUC of each G-protein predictor) and dividing by the number of classes. The micro average is calculated by aggregating all individual predictions/ground truth labels from all predictors and computing the ROC AUC on the aggregated set, as if they were a single task.

##### Validating the design choices in the machine learning method

Our machine learning method incorporates the use of orthologs to augment the training set of pre-trained sequence embeddings from ProTrans and the subsequent neural networks (NN). In order to validate the effect of the data augmentation on the performance of the method, we attempted a number of variants to evaluate their effect on performance (Figure S3). Specifically, we tested our method (termed NN+pretrained+orthologs) vs. a variant with no orthologs during training (NN + pretrained), vs. a variant with no pretrained embeddings (NN + orthologs), and a variant using a simpler machine learning technique, logistic regression, instead of an NN (logistic+orthologs).

#### Deep learning model predictions of G protein selectivity

To test our hypothesis that our method could predict the G protein coupling selectivity of GPCRs in Class A GPCRs, we selected a set of 8 previously uncharacterized Class A GPCRs from *Drosophila*. These served as our prospective prediction set. Simultaneously, we selected 94 already characterized Class A GPCRs (Figure 1) to act as our learning set.

The learning process in deep learning operates in cycles, commonly known as epochs. Training continues until a satisfactory level of progress has been achieved, with the optimal neural network saved at the conclusion of each epoch. Thus, to assess the network’s performance and select the best network, it is necessary to partition our learning set into a training set (containing 84 GPCRs) and a validation set (containing 10 GPCRs). The training set aids the machine learning algorithm in learning the target function, while the validation set serves to confirm the learning process after each epoch.

It’s essential to note that the method in which the learning set is split into training and validation sets can influence the performance of the model because the algorithm is learning from a different set of GPCRs in each partition. Consequently, we carried out 10 runs using randomly sampled training and validation sets for the prediction of our 8 Class A prospective GPCRs.

For each Class A GPCR, we computed a confidence score for our prediction using a technique called Monte Carlo Dropout (https://proceedings.mlr.press/v48/gal16.html?trk=public_post_comment-text). This method allowed us to estimate the predictive uncertainty in our deep learning model. In simple terms, Monte Carlo Dropout works by randomly ‘dropping out’ or deactivating certain neurons during training, creating a ‘forest’ of different neural networks. During prediction, we run our model many times, each time with a different ‘drop out’ configuration, which gives us a range of different predictions. The variance in these predictions gives us an estimate of the uncertainty or confidence in our predictions.

After performing this Monte Carlo Dropout, we assigned to each prospective prediction the value in the neural network with the highest computed confidence, thus finalizing our predictive model for the coupling preferences of G proteins to our set of 8 GPCRs.

#### Common numbering systems of GPCRs and Gα proteins

Each amino acid of GPCRs and Gα subunits was assigned a number according to the common numbering systems used in GPCRdb (https://gpcrdb.org/) so that its arrangement in the protein structure would be reflected (Figure S3). It is important to note, however, that a slight modification was made to the intracellular loops of the GPCR. The amino acids in ICL1 and ICL2 were manually corrected according to the sequence conservation. A common numbering has not been established for ICL3 because of its wildly diverse length and lack of sequence homology. Because the regions of ICL3 that bind Gα are confined to the region near TM5 and TM6, ICL3 has been divided in half and numbered, so that the numbers can be counted from the N- and C-termini inward (Table S3). In this study, only TMs and ICLs involved in Gα binding were analyzed using the common numbering system, and extracellular domains not involved in Gα binding were not considered.

#### Residue-residue networks of GPCR-Gα interactions

Structural data for 33 GPCR/Gα complexes, including 19 GPCR/G_i/o_ complexes, 3 GPCR/G_q_ complexes, and 11 GPCR/G_s_ complexes, were retrieved from the Protein DataBank (https://www.rcsb.org/). Of note, if there was more than one structure available for a given GPCRs, the one with the highest resolution was used for our analysis. The PDB numbers of the structures used in the structural analysis can be found in Table S2. Meanwhile, the structural data of the GPCR/G_q_ complexes (6WHA, 7L1U, and 7MBY) using mini-G_qsi_ instead of Gα_q_ was excluded from the analysis because the protein sequence of mini-G_qsi_ is heavily modified with amino acids derived from non-Gα_q_ sequences.

GPCR-Gα interactions were analyzed using bioCOmplexes COntact MAPS (COCOMAPS, http://www.molnac.unisa.it/BioTools/cocomaps/) with a cutoff value of 5Å for identifying interacting residues in both molecules.^69^ The amino acid interaction maps for each complex were mapped (Table S2) using the common numbering systems. A total of 106 positions were involved in Gα binding at least once in the complexes used for this analysis (Figure 4B). These residues were referred as the Gα-binding residues with one exception. Because it was a highly specific interaction only found in rhodopsin with highly specific photoreceptor function, the residues of rhodopsin’s C-terminal tail that interact with Gα were excluded from the Gα-binding residues.

From the integrated residue-residue contacts between class A GPCRs and Gα subunits identified by our analysis (Figure 4E), we further extracted contacts common to all GPCRs and Gα subunits and contacts used by each type of Gα subfamily. As a first step, we divided the GPCR/G protein complexes by interacting G protein subfamilies (G_i/o_, G_q_, vs. G_s_). Then, we defined a common residue-residue network as residue-residue contacts that occur at least once in each three classes of GPCR/G protein complexes (GPCR/G_i/o_, GPCR/G_q_, and GPCR/G_s_ complexes) (Figure 4F). The specific residue-residue contacts for G_i/o_, G_q_, or G_s_ interactions are defined by residue-residue interaction pairs present only with G_i/o_, G_q_, or G_s_ (Figures 4G–4I).

#### Sankey diagram

Using a web-based drawing tool (https://sankeymatic.com/), Sankey diagrams were created to visualize the interactions between different segments of GPCRs and G proteins (Figures 4E–4M), as well as the rank order of G protein selectivity (Figure 2E).

#### Protein sequence alignment and phylogenetic tree

The alignment of the protein sequences of Gα subunits (Figure S4) was performed using the multiple sequence alignment tool, ClustalW (https://www.genome.jp/tools-bin/clustalw). The phylogenetic tree was drawn using FigTree v1.4.4. software. The branches of the phylogenetic tree were color-coded based on the Gα subfamily (Figure 1E) or the type of ligand (Figures 4N and S5).

#### Analysis of natural genetic variants

Natural genetic variants for 284 genes of class A GPCR are retrieved from the Genome Aggregation Database (gnomAD) v2.1.1 (https://gnomad.broadinstitute.org/),^48^ which consists of genome and exome sequence data for 141,456 human subjects in seven ethnic groups. After converting the amino acid numbers of the GPCRs to the common numbering, the missense variants in the Gα-binding residues were extracted and analyzed. The number of distinct missense variants, number of positions with missense variants, and density of missense variants were calculated for each gene. Based on the *Z* score, each position was analyzed to determine whether it contained more or fewer MVs in comparison to the average number of MVs present in the Gα-binding residues (Figure 6E). Figure S5D shows that accurate *Z* score cannot be obtained if there are fewer than 19 GPCRs in each position, such positions are not included for this analysis. Since the number of GPCRs with each position is different, we first normalized the number of mutants in each position by dividing it by the number of receptors. *Z* score is calculated using the formula z = (x-μ)/σ, where x is the raw score, μ is the population mean, and σ is the population standard deviation.

### QUANTIFICATION AND STATISTICAL ANALYSIS

two-way ANOVA with correction for multiple comparison using the Sidak method was conducted to determine if GPCRs can activate G proteins and the rank order of G protein selectivity with GraphPad Prism Ver. 6. Only statistically significant values are reported. Values represent means ± SEM from three independent experiments each performed with at least three replicates.

## Supplementary Material

1

2

3

4

5

## Figures and Tables

**Table:** KEY RESOURCES TABLE

REAGENT or RESOURCE	SOURCE	IDENTIFIER
Chemicals, peptides, and recombinant proteins		

Dulbecco’s modified Eagle’s medium	Thermo Fisher Scientific	11965-092
Fetal bovine serum	Genesee Scientific	25-550
Sodium pyruvate	Thermo Fisher Scientific	11360-070
MEM non-essential amino acids	Thermo Fisher Scientific	11140-050
Penicillin-streptomycin	Thermo Fisher Scientific	15140-122
Matrigel	Corning	356230
Lipofectamine LTX and Plus reagent	Thermo Fisher Scientific	15338-100
Dulbecco’s phosphate-buffered saline	MilliporeSigma	D5652
Neurobasal-A Medium	Thermo Fisher Scientific	10888-022
GlutaMAX	Thermo Fisher Scientific	35050-061
B-27 Supplement	Thermo Fisher Scientific	17504-044
DNAse I	Thermo Fisher Scientific	18047019
Poly-D-lysine hydrobromide	MilliporeSigma	P6407
Papain	Worthington Biochemical	LS003126

Critical commercial assays		

Nano-Glo Luciferase Assay Substrate (furimazine)	Promega	N1120

Experimental models: Cell lines		

HEK293T/17	ATCC	CRL-11268

Recombinant DNA		

GPR52	cDNA Resource Center	GPR0520000
HTR1A	cDNA Resource Center	HTR01A0000
HTR1B	cDNA Resource Center	HTR01BTN00
HTR1D	cDNA Resource Center	HTR01D0000
HTR1E	cDNA Resource Center	HTR01E0000
HTR1F	cDNA Resource Center	HTR01F0000
HTR2A	cDNA Resource Center	HTR02A0001
HTR2B	cDNA Resource Center	HTR02B0000
HTR2C	cDNA Resource Center	HTR02C0000
HTR4	cDNA Resource Center	HTR040000B
HTR5A	cDNA Resource Center	HTR05A0000
HTR6	cDNA Resource Center	HTR0600000
HTR7	cDNA Resource Center	HTR07A0000
HTR7	cDNA Resource Center	HTR07B0000
CHRM1	cDNA Resource Center	MAR0100000
CHRM2	cDNA Resource Center	MAR0200000
CHRM3	cDNA Resource Center	MAR0300000
CHRM4	cDNA Resource Center	MAR0400000
CHRM5	cDNA Resource Center	MAR0500000
ADRA1A	cDNA Resource Center	AR0A1A0001
ADRA1B	cDNA Resource Center	AR0A1B0000
ADRA1D	This study	N/A
ADRA2A	cDNA Resource Center	AR0A2A0000
ADRA2B	cDNA Resource Center	AR0A2B0000
ADRA2C	cDNA Resource Center	AR0A2C0000
ADRB1	This study	N/A
ADRB2	cDNA Resource Center	AR0B200000
ADRB3	cDNA Resource Center	AR0B300000
DRD1	cDNA Resource Center	DRD0100000
DRD2	cDNA Resource Center	DRD0200001
DRD3	cDNA Resource Center	DRD03A0000
DRD4	cDNA Resource Center	DRD0400000
DRD5	cDNA Resource Center	DRD0500000
HRH1	cDNA Resource Center	HRH0100000
HRH2	cDNA Resource Center	HRH0200000
HRH3	cDNA Resource Center	HRH0300000
MTNR1A	cDNA Resource Center	MTNR1A0000
MTNR1B	cDNA Resource Center	MTNR1B0000
ADORA1	cDNA Resource Center	ADRA100000
ADORA2A	cDNA Resource Center	ADRA2A0000
ADORA2B	cDNA Resource Center	ADRA2B0000
ADORA3	cDNA Resource Center	ADRA300000
GALR1	cDNA Resource Center	GALR100000
GALR2	cDNA Resource Center	GALR200000
GALR3	cDNA Resource Center	GALR300000
OPRM1	cDNA Resource Center	OPRM100000
OPRD1	cDNA Resource Center	OPRD100000
OPRK1	cDNA Resource Center	OPRK100000
OPRL1	cDNA Resource Center	OPRL100000
EDNRA	cDNA Resource Center	EDNRA00000
EDNRB	cDNA Resource Center	EDNRB00000
GRPR	cDNA Resource Center	GRPR000000
BRS3	cDNA Resource Center	BRS0300000
APLNR	cDNA Resource Center	AGTL100000
AVPR1A	cDNA Resource Center	AVR01A0000
AVPR1B	cDNA Resource Center	AVR01B0000
AVPR2	cDNA Resource Center	AVR0200000
BDKRB2	cDNA Resource Center	BDKB200000
MC1R	cDNA Resource Center	MCR0100000
MC3R	cDNA Resource Center	MCR0300000
MC4R	cDNA Resource Center	MCR0400000
MC5R	cDNA Resource Center	MCR0500000
NPY1R	cDNA Resource Center	NPYR100000
NPY2R	cDNA Resource Center	NPYR200000
NPY4R	cDNA Resource Center	NPYR400000
NPY5R	cDNA Resource Center	NPYR500000
TACR1	cDNA Resource Center	TACR100000
TACR2	cDNA Resource Center	TACR200000
TACR3	cDNA Resource Center	TACR300000
NTSR1	cDNA Resource Center	NTSR100000
HCRTR1	cDNA Resource Center	HCR0100000
HCRTR2	This study	N/A
OXTR	cDNA Resource Center	OXTR000000
CCKAR	cDNA Resource Center	CCKAR00000
CCKBR	cDNA Resource Center	CCKBR00000
NPFFR1	cDNA Resource Center	NPFFR10000
SSTR1	cDNA Resource Center	SSTR100000
SSTR2	cDNA Resource Center	SSTR200000
SSTR3	cDNA Resource Center	SSTR300000
SSTR4	cDNA Resource Center	SSTR400000
SSTR5	cDNA Resource Center	SSTR500000
KISS1R	cDNA Resource Center	KISS1R0000
CXCR4	cDNA Resource Center	CXCR400000
PTGER1	cDNA Resource Center	PER0100000
PTGER2	cDNA Resource Center	PER0200000
PTGER3	This study	N/A
PTGER4	cDNA Resource Center	PER0400000
PTGDR	cDNA Resource Center	PTGDR00000
PTGDR2	cDNA Resource Center	CRTH200000
TBXA2R	cDNA Resource Center	TXA2R00000
CNR1	cDNA Resource Center	CNR0100001
GPBAR1	This study	N/A
SUCNR1	cDNA Resource Center	SUCNR10000
OXGR1	cDNA Resource Center	OXGR100000
GCGR	cDNA Resource Center	GCGR000000
GIPR	This study	N/A
GHRHR	cDNA Resource Center	GHRHR00000
GLP1R	This study	N/A
GLP2R	cDNA Resource Center	GLP2R00000
SCTR	cDNA Resource Center	SCTR000000
CALCR	cDNA Resource Center	CALCR00000
CALCRL	cDNA Resource Center	CALCRL0000
PTH1R	cDNA Resource Center	PTHR100000
PTH2R	cDNA Resource Center	PTHR200000
VIPR1	cDNA Resource Center	VIPR100000
VIPR2	cDNA Resource Center	VIPR200000
PACAPR	cDNA Resource Center	ACP1R10000
CRHR1	cDNA Resource Center	CRHR100000
CRHR2	cDNA Resource Center	CRHR200000
GRM1	This study	N/A
GRM2	cDNA Resource Center	GRM2000000
GRM3	cDNA Resource Center	GRM3000000
GRM4	cDNA Resource Center	GRM4000000
GRM5	This study	N/A
GRM6	cDNA Resource Center	GRM6000000
GRM7	cDNA Resource Center	GRM7000001
GRM8	cDNA Resource Center	GRM8000000
GABBR1	This study	N/A
GABBR2	cDNA Resource Center	GABBR20000
hM4D(Gi)	Addgene	45548
hM3D(Gq)	Addgene	45547
rM3D(Gs)	Addgene	45549
G12D 1.0	Dr. Asuka Inoue	N/A
G12D 2.0	Dr. Asuka Inoue	N/A
G12D 2.1	Dr. Asuka Inoue	N/A
mAChR-A (Drosophila)	This study	N/A
mAChR-B (Drosophila)	This study	N/A
Dop1R1 (Drosophila)	This study	N/A
Dop2R (Drosophila)	This study	N/A
DAMB (Drosophila)	Dr. Ronald L. Davis	N/A
5HT1A (Drosophila)	This study	N/A
5HT1B (Drosophila)	This study	N/A
5HT2A (Drosophila)	This study	N/A
5HT2B (Drosophila)	This study	N/A
MRAP	This study	N/A
RAMP1	cDNA Resource Center	RAMP100000
Gα_oA_	cDNA Resource Center	GNA0OA0000
Gα_q_	Dr. Hiroshi Itoh	N/A
Gα_15_	cDNA Resource Center	GNA1500000
Gα_s_ short isoform	Dr. Hiroshi Itoh	N/A
Gα_13_	cDNA Resource Center	GNA1300001
Venus-156-239-Gβ_1_	Dr. Nevin A. Lambert	N/A
Venus-1-155-Gγ_2_	Dr. Nevin A. Lambert	N/A
masGRK3ct-Nluc-HA	Masuho et al.^27^	N/A
Flag-Ric-8A	Dr. Jean-Pierre Montmayeur	N/A
Flag-Ric-8B	Dr. Bettina Malnic	N/A
PTX-S1	Raveh et al.^61^	N/A
DORA-RhoA (BRET)	This study	N/A
CalfluxVTX	Yang et al.^62^	N/A
Gα_oA_-Nluc	Masuho et al.^27^	N/A
p115RH-AU5-CAAX	This study	N/A
pENN.AAV.hSyn.Cre.WPRE.hGH	Addgene	105553

Software and algorithms		

GraphPad Prism 9	GraphPad Software	https://www.graphpad.com/
SigmaPlot 14.5	SYSTAT Software	https://systatsoftware.com/
Clampfit 10.3	Molecular Devices	http://www.moleculardevices.com/products/software/pclamp.html

## Highlights

- Systematic analysis of GPCR enzymatic activity unveils G protein selectivity across 124 GPCRs
- Functional rank-order-based classification for GPCRs is introduced
- Machine learning accurately predicts GPRC-Gα selectivity
- Natural genetic variation in GPCRs influences their G protein preferences
