# Supplementary material for: Rules and mechanisms governing G protein coupling selectivity of GPCRs
Source: Cell Rep. Author manuscript; Available in PMC 2024 Feb 5. (PMC10842385; doi:10.1016/j.celrep.2023.113173)
Supplement: 1 [file NIHMS1941869-supplement-1.pdf]

Cell Reports, Volume *42*

Supplemental information

Rules and mechanisms governing G protein coupling selectivity of GPCRs

Ikuo Masuho, Ryoji Kise, Pablo Gainza, Ee Von Moo, Xiaona Li, Ryosuke Tany, Hideko Wakasugi-Masuho, Bruno E. Correia, and Kirill A. Martemyanov

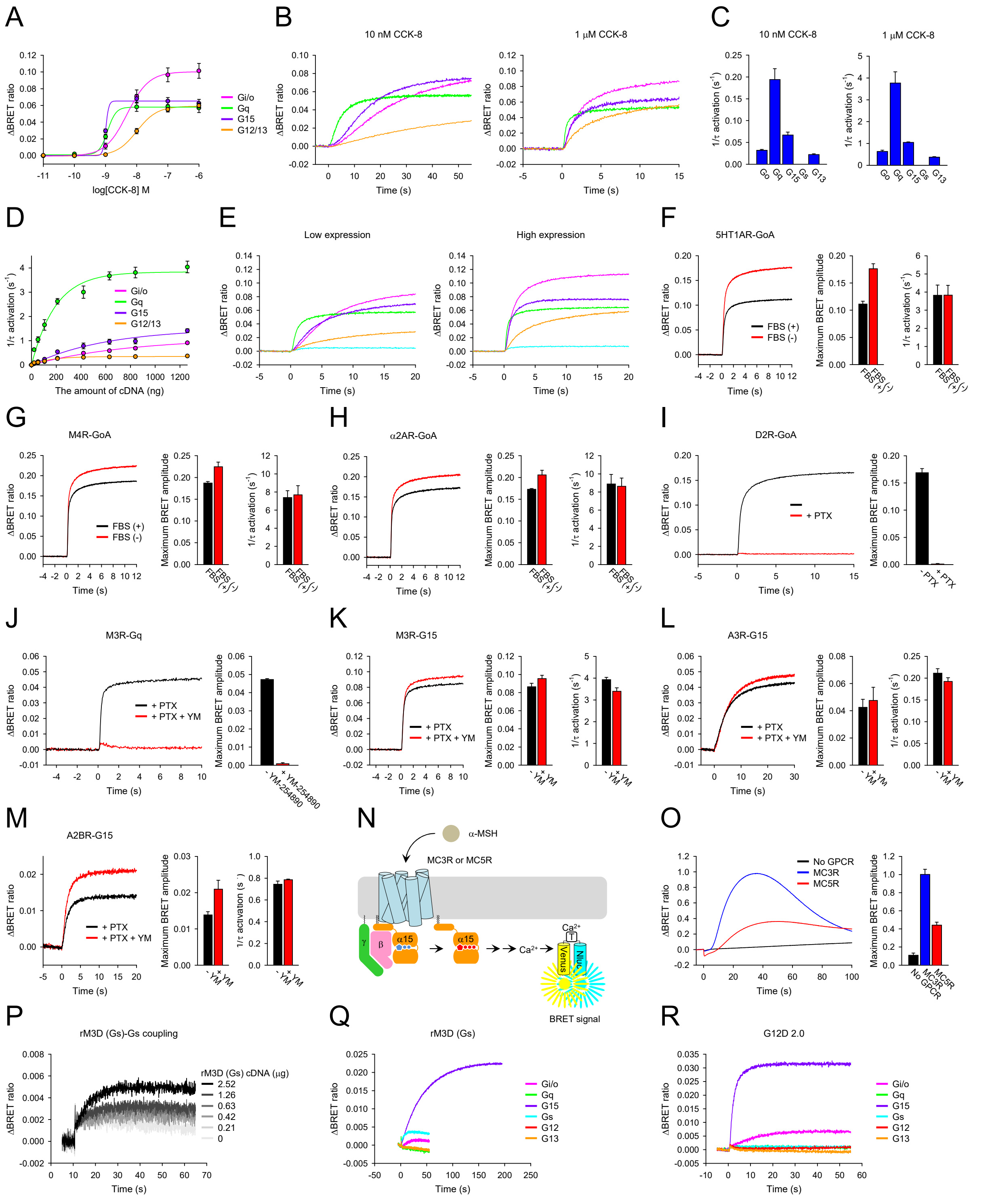

Figure S1 (Related to Figure 1). The effect of agonist concentration and GPCR expression level on the G protein coupling profile. (**A**) Dose-response analysis of CCK$_2$R with four G proteins. (**B**) Time-course of G protein activation by CCK$_2$R stimulated by 10 nM and 1 μM CCK-8. (**C**) The activation rates quantified from the panel B were plotted as bar graphs. (**D**) The correlation between the amount of CCK$_2$R cDNA used for transfection and the rate of G protein

activation induced by saturating concentrations of agonists. (**E**) Time-course of G protein activation at low and high expression levels of CCK_2R. (**F-H**) Impact of serum starvation on the activity of $5HT_{1A}R$, M_4R, and $\alpha_{2A}R$. (**I-M**) Confirming the G_{15} coupling with the treatment of PTX and YM-254890 (YM). The expression of PTX-S1 inhibited G_{oA} activation (**I**). The treatment of transfected cells with 10 μM YM for 5 min inhibited G_q activation (**J**) but not G_{15} activation (**K**). The treatment of transfected cells with PTX and YM did not inhibit G_{15} activation by adenosine A3 receptor (A_3R) (**L**) or adenosine A2B receptor ($A_{2B}R$) (**M**), confirming their specific coupling. (**N**) Schematic representation of the BRET-based assay for real-time monitoring of intracellular Ca^{2+} concentration using CalfluxVTN sensor. Ca^{2+} bind to the Troponin C domain (T), causing a conformational change that leads to an increase in the BRET signal. (**O**) G_{15}-coupling of MC_3R and MC_5R. (**P**) Optimizing the amount of rM3R (Gs) cDNA for transient transfection. The highest amount of the cDNA was used to examine the G protein coupling profile of rM3R (Gs) in panel Q. (**Q**) Time-course of G protein activation by rM3D (Gs). (**R**) Time-course of G protein activation by G12D2.0. For traces, the average values of the three independent experiments are shown. In bar graphs and scattered plots, the average values ± SEM of the three independent experiments are shown.

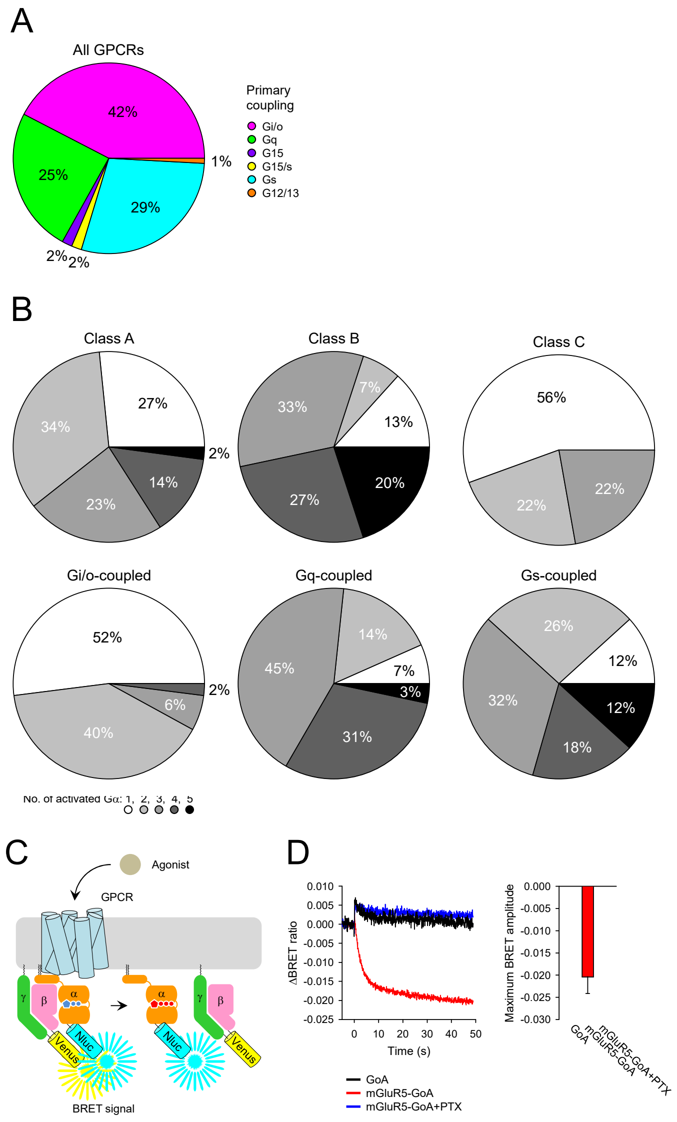

Figure S2 (Related to Figure 2). The primary coupling and promiscuity of GPCRs tested in this study. (**A**) The primary G protein coupling selectivity of all GPCRs. (**B**) The promiscuity of GPCRs by sequence-based class (class A, B, *vs*. C) and function-based class ($G_{i/o}$-, G_q-, vs. G_s-coupled receptors). (**C**) Schematic representation of the BRET assay for G protein activation using dissociation of $G\alpha_{oA}$ and Gβγ as readout. GPCR stimulation by agonist dissociates Venus-Gβγ and $G\alpha_{oA}$-Nluc and decreases the BRET ratio. (**D**) Agonist induced-mGluR5 dependent G_{oA} activation. Stimulation of mGluR5 with 1 mM L-glutamic acid induced G_{oA} activation. This activation was inhibited by PTX-S1 expression, confirming the coupling of mGlur5 and G_{oA}. The average values of the three independent experiments are shown (**D**). The average values ± SEM of the three independent experiments are shown (**D**).

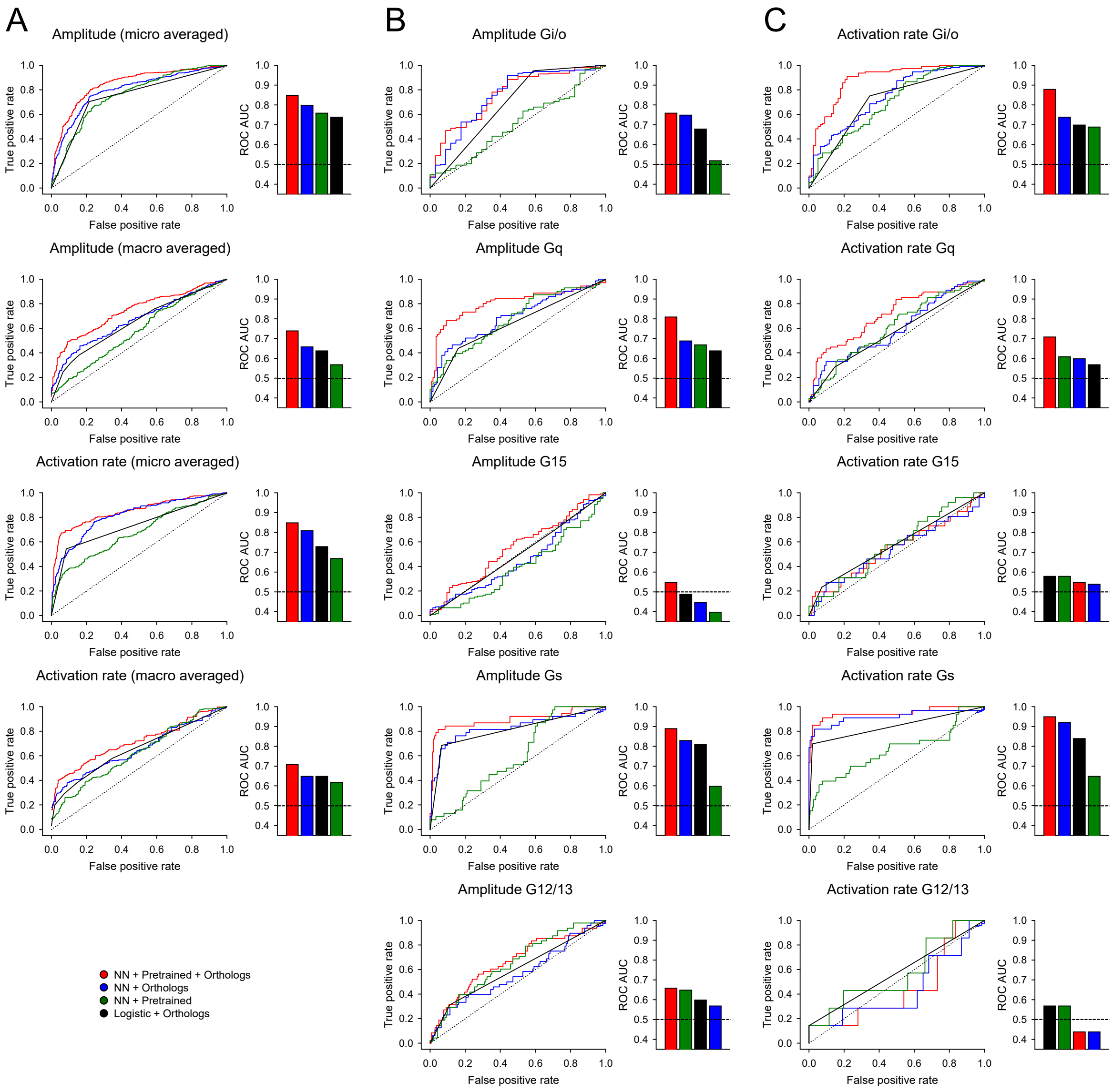

Figure S3 (Related to Figure 3). Validation of the machine learning-based prediction techniques for G protein coupling selectivity. (**A**) A micro average or a macro average of the ROC AUC for amplitude- or activation rate-based pridectors. (**B and C**) Per-G protein subfamily performance for amplitude (**B**) or activation rate (**C**). Three of variants of our methods were tested to validate the method's design decisions: NN+pretrained+orthologs (our method (red)), NN+orthologs (our method trained/tested with no pretrained embeddings (blue)), NN+pretrained (our method trained with no orthologs (green)), logistic+orthologs (our method trained with a logistic regression and no pretrained embeddings instead of a neural network(black)) (**A-C**).

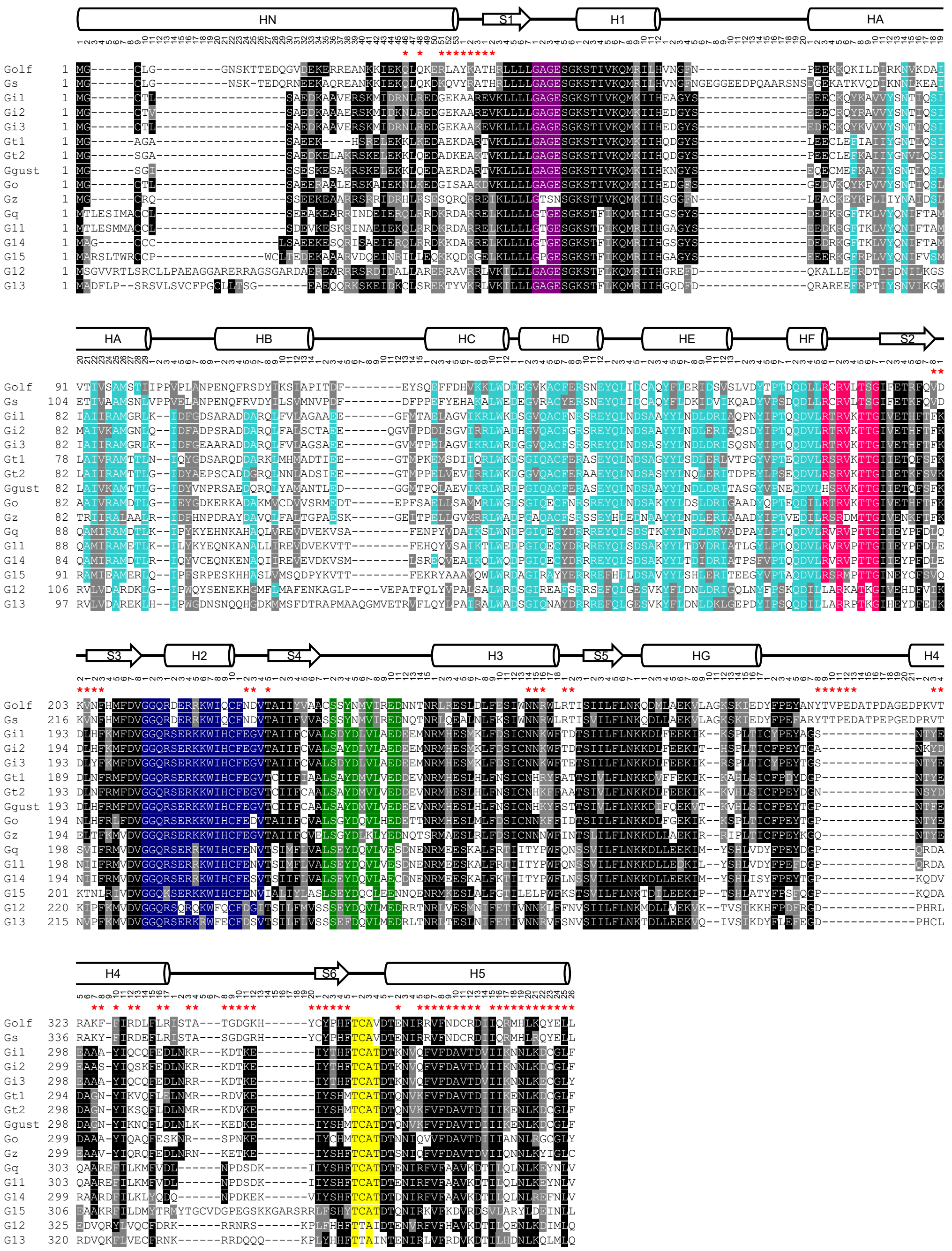

Figure S4 (Related to Figure 4). A common numbering of Gα subunits with GPCR-binding residues. The GPCR-binding residues are highlighted by red asterisks.

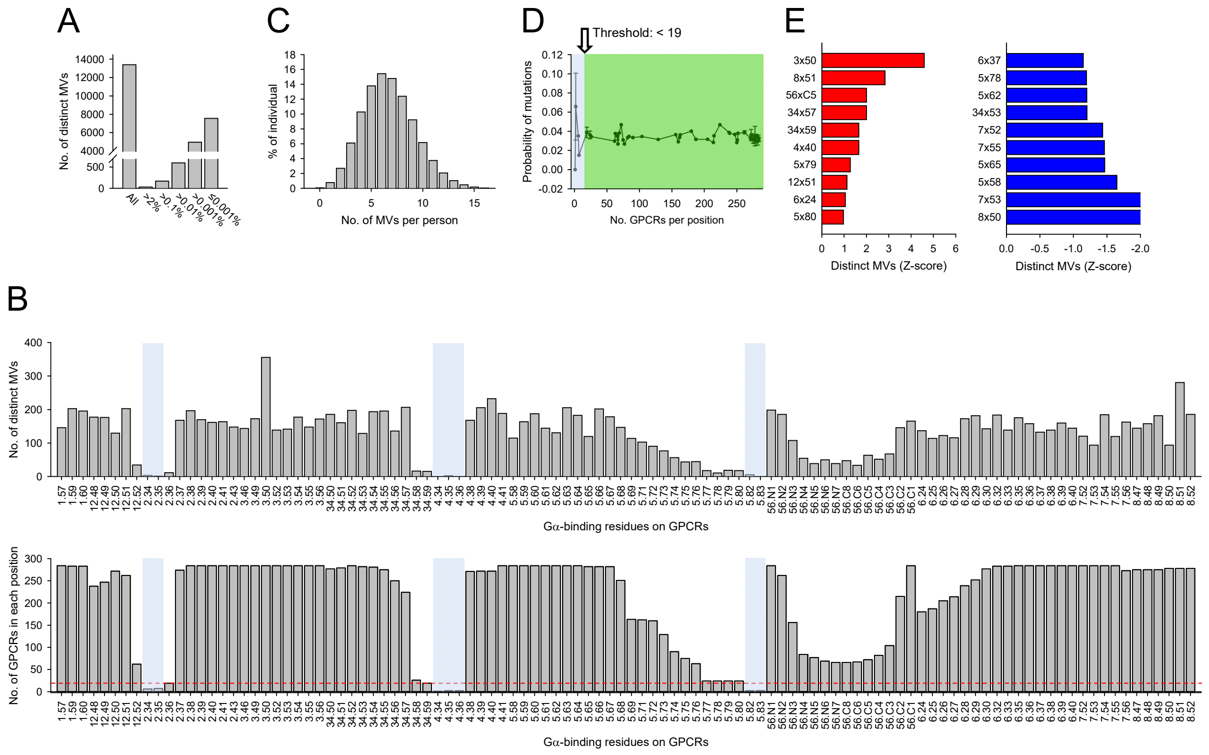

Figure S5 (Related to Figure 6). The number of MVs in the Gα-binding residues of class A GPCRs. (**A**) The number of unique MVs in Gα-binding residues per frequency. This analysis indicated that most of MVs in Gα-binding residues are rare (<2%), suggesting that these variations may be related to individual non-disease traits. (**B**) The number of distinct MVs in each position of Gα-binding residue and the number of GPCRs possessing each position. The red dotted line indicates the threshold identified in panel C. (**C**) The probability of MVs present in Gα-binding residue per the number of GPCRs. Based on the number of GPCRs at each position and the possibility of 19 amino acids entering, the probability was calculated. We set the threshold at 19 because probabilities stabilize around 0.04 when there are more than 19 GPCRs. (**D**) The ranking of Gα-binding positions by Z score.

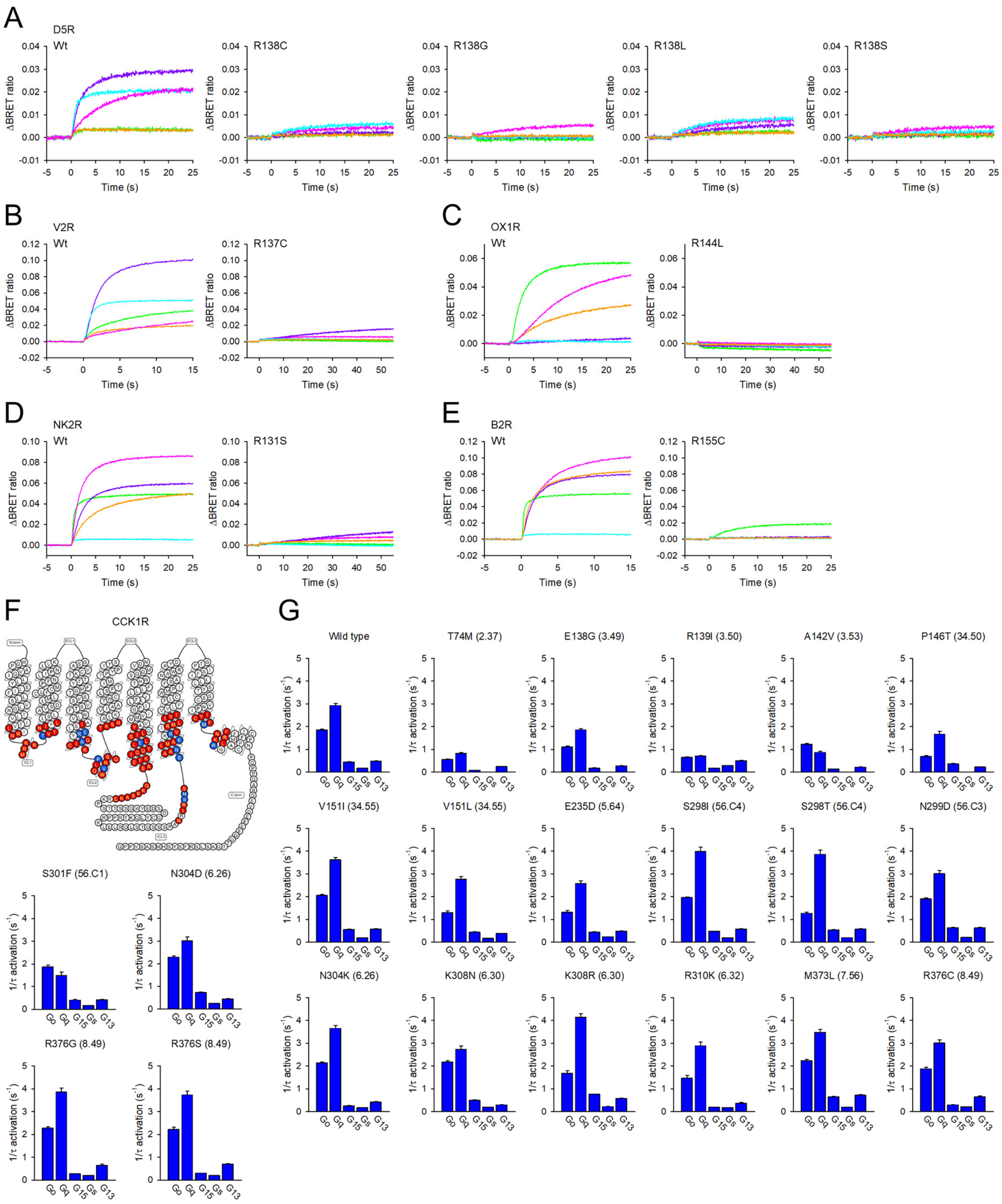

Figure S6 (Related to Figure 6). Functional characterization of MVs in the Gα-binding residues of class A GPCRs. (**A-E**) Functional characterization of MVs in 3.50 of D_1R (**A**), V_2R (**B**), OX_1R (**C**), NK_2R (**D**), and B_2R (**E**). (**F**) The snake plot of CCK_1R with the Gα-binding residue (red) and positions of characterized MVs (blue). (**G**) The activation rates of wild type and mutant CCK_1Rs. The average values of the three independent experiments are shown (**A-E**). The average values ± SEM of the three independent experiments are shown (**G**).
